# Immobilization of Cr(VI) by sulphate green rust and sulphidized nanoscale zerovalent iron in sand media: batch and column studies

**DOI:** 10.1186/s12932-020-00073-9

**Published:** 2020-08-14

**Authors:** Flavia Digiacomo, Dominique J. Tobler, Thomas Held, Thomas Neumann

**Affiliations:** 1 ARCADIS Germany GmbH, Griesbachstraße 10, 76185 Karlsruhe, Germany; 2 Institute of Applied Geosciences, Karlsruhe Institute of Technology, Adenauerring 20b, Building 50.40, 76131 Karlsruhe, Germany; 3 Nano-Science Center, Department of Chemistry, University of Copenhagen, Universitetsparken 5, 2100 Copenhagen, Denmark; 4 ARCADIS Germany GmbH, Europaplatz 3, 64293 Darmstadt, Germany; 5 Department of Applied Geosciences, Technical University of Berlin, Ernst-Reuter-Platz 1, 10587 Berlin, Germany

**Keywords:** Sulphate green rust, Sulphidized nZVI, Hexavalent chromium, Chromate, Packed bed columns, Reduction, Remediation

## Abstract

Chromate, Cr(VI), contamination in soil and groundwater poses serious threat to living organisms and environmental health worldwide. Sulphate green rust (GR_SO4_), a naturally occurring mixed-valent iron layered double hydroxide has shown to be highly effective in the reduction of Cr(VI) to poorly soluble Cr(III), giving promise for its use as reactant for in situ remedial applications. However, little is known about its immobilization efficiency inside porous geological media, such as soils and sediments, where this reactant would ultimately be applied. In this study, we tested the removal of Cr(VI) by GR_SO4_ in quartz sand fixed-bed column systems (diameter × length = 1.4 cm × 11 cm), under anoxic conditions. Cr(VI) removal efficiency (relative to the available reducing equivalents in the added GR_SO4_) was determined by evaluating breakthrough curves performed at different inlet Cr(VI) concentrations (0.125–1 mM) which are representative of Cr(VI) concentrations found at contaminated sites, different flow rates (0.25–3 ml/min) and solution pH (4.5, 7 and 9.5). Results showed that (i) increasing Cr(VI) inlet concentration substantially decreased Cr(VI) removal efficiency of GR_SO4_, (ii) flow rates had a lower impact on removal efficiencies, although values tended to be lower at higher flow rates, and (iii) Cr(VI) removal was enhanced at acidic pH conditions compared to neutral and alkaline conditions. For comparison, Cr(VI) removal by sulphidized nanoscale zerovalent iron (S-nZVI) in identical column experiments was substantially lower, indicating that S-nZVI reactivity with Cr(VI) is much slower compared to GR_SO4_. Overall, GR_SO4_ performed reasonably well, even at the highest tested flow rate, showing its versatility and suitability for Cr(VI) remediation applications in high flow environments.
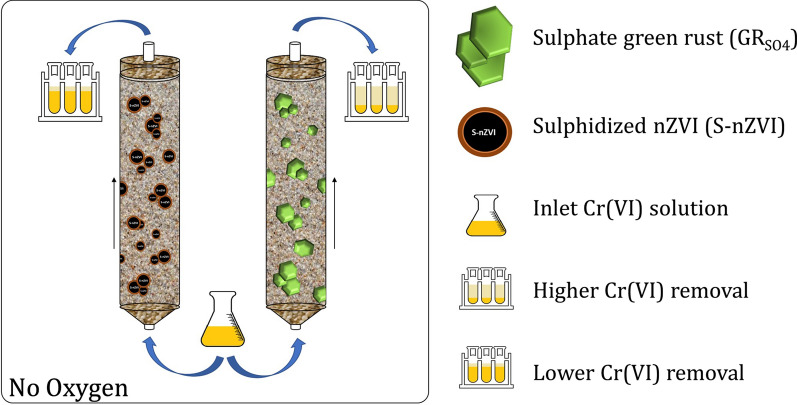

## Introduction

Chromium (Cr) is one of the most common heavy metals found in the biosphere and a key micronutrient, but it is also a frequent contaminant in soils and groundwater worldwide, due to the uncontrolled release of Cr contaminated waters from various industries (e.g., wood treatment, electroplating operations, leather tanning or metal plating solutions). Chromium mainly exists in two oxidation states under near surface conditions: the trivalent form, Cr(III), and the hexavalent form, Cr(VI). Cr(III) is highly insoluble, generally nontoxic [1], and thus of low concern. Moreover, Cr(III) species [i.e., Cr(OH)^2+^, or Cr(OH)_2_^+^] are positively charged at acidic and near neutral groundwater pHs, meaning they are easily adsorbed onto negatively charged soil particles [2], such as quartz grains and clay minerals. In contrast, the dominant Cr(VI) species at pH > 7 is chromate (CrO_4_^2−^), whilst at pH values between 2 and 7, Cr(VI) may be present as dichromate (Cr_2_O_7_^2−^) and hydrogen chromate (HCrO_4_^−^). These negatively charged Cr(VI) oxyanions act as strong oxidants, are highly soluble (i.e., the dominant species in industry waste spills), are known to be highly toxic, mutagenic and carcinogenic and thus pose severe threats to ecosystems and human health. In many countries, it is therefore a key mandate to clean-up Cr contaminated sites.

An efficient way to clean-up Cr(VI) contaminated waters is by reaction with reductant materials, which most often consist of sulphur and/or iron bearing materials, e.g., sodium dithionite, iron sulphides, dissolved Fe(II) [3–5], magnetite [6], and zerovalent iron (ZVI) [7–9]. In this process, Cr(VI) is quickly reduced to Cr(III), which leads to instant immobilisation as insoluble Cr(III) phases (e.g., Cr(OH)_3_ or Cr-bearing iron oxyhydroxides [10]) due to their low solubility.

Pump-and-treat (P&T) is a common ex situ method to clean up groundwater contaminants. In this approach, groundwater is extracted and treated above ground prior to discharge. The extraction design (number and location of extraction wells) and the treatment system (i.e., need of a holding tank, single or multiple clean-up method) are strongly dependent on the local hydrogeochemical conditions, which can make installation and running thereof difficult, particularly in urban areas. Moreover, P&T facilities have to deal with large amounts of wastes produced as a result of treatment [i.e., Cr(III)-bearing sludge or Cr(III) plugged filters] and treatment times are very long (e.g., 50–100 years); thus, clean-up goals are often difficult to reach with P&T. Faster and less disruptive clean-up is obtained by in situ treatment via emplacement of reactants in the subsurface to create permeable reactive barriers (PRBs) or zones, where contaminants are degraded and/or immobilised, often under reducing conditions.

Nanoscale ZVI (nZVI) is frequently applied for in situ treatment of subsurface Cr(VI) contamination, however, it is non-selective and quickly reacts with water and other oxidants and thereby loses reactivity quickly [11–13]. Sulphidation of nZVI has recently emerged as a new approach to counterbalance some of these disadvantages [14], whereby an FeS shell is created around the metallic iron core, to protect the core from anoxic corrosion and rapid loss of reducing equivalents. Sulphidized-nZVI (S-nZVI) has shown to be more reactive with Cr(VI) compared to non-sulphidized nZVI, both in anoxic [15] and oxic systems [16]. However, there are also materials such as green rusts (GRs) which are equally, if not more reactive with Cr(VI), representing a promising alternative to nZVI. GRs are natural occurring Fe(II)/Fe(III) layered double hydroxides (LDHs) that often form in suboxic/anoxic, Fe^2+^-rich environments [17, 18]. GRs have been increasingly investigated over the past 20 years due to their high reactivity with Cr(VI) [19–23], as well as many other contaminants, including NO_2_^−^ [24], NO_3_^−^ [25], Se(VI) [26, 27], U(VI) [28], tetra- and trichloromethane [29] and nitroaromatic compounds [30, 31].

GR materials consist of positively charged Fe(II)/Fe(III) hydroxide layers, that sandwich negatively charged, hydrated interlayers containing anions (e.g., SO_4_^2−^, CO_3_^2−^ and Cl^−^) [32, 33] and occasionally cations [34]. Besides its natural occurrence, GR is frequently observed as a corrosion product of steel in O_2_-limited settings [35] and metallic iron in PRBs [36–38]. Of all green rust types, sulphate-bearing green rust (GR_SO4_) is the most commonly used GR material in Cr(VI) reduction studies, most likely due to its ease of synthesis and because CrO_4_^2−^ and SO_4_^2−^ anions have similar tetrahedral structure and charge which allows CrO_4_^2−^ to diffuse into the GR_SO4_ interlayer [39, 40]. Several batch studies have determined Cr(VI) reduction rates by GR_SO4_ and combined these with the characterization of the oxidation end-products to determine reaction kinetics and the fate of the formed Cr(III) [19–23]. All these studies have demonstrated that reduction rates are extremely fast, with most Cr(VI) reduced within the first 10 min of the reaction. However, the type of oxidation product can vary depending on the applied Cr(VI)/Fe(II) ratio [23], the presence of aqueous Fe(II) [19], GR type [20–22, 41, 42] as well as GR synthesis and preparation protocols (e.g., fresh, washed and/or aged GR) [19]. These batch studies have provided key information on GR-Cr(VI) interactions and these certainly help towards prediction of GR effectiveness in Cr(VI) wastewater tanks (e.g., pump-and-treat facilities). However, observations from batch solution studies give little insight into behaviour in porous subsurface environments as would be needed to predict GR effectiveness in PRBs. In these settings, GR will be exposed to dynamic flow conditions and steady Cr(VI) concentrations, while it may also interact with surrounding grain surfaces. So far, no study has looked at GR reactivity with Cr(VI) in sand matrices, whether in batch nor column systems but such information is critically needed to evaluate the potential use of GR as alternative reductant material to ZVI/S-nZVI in in situ subsurface treatment applications such as PRBs.

This study assesses Cr(VI) immobilisation by synthetic sulphate green rust (GR_SO4_) under dynamic flow conditions inside packed sand columns. Specifically, we tested the impact of flow rate, inlet Cr(VI) concentration, and pH conditions on Cr(VI) removal efficiency by evaluation of breakthrough curves. In addition, we also examined Cr(VI) reduction by GR_SO4_ in batch sand experiments (static conditions) for comparison of removal efficiencies to experiments under dynamic flow. Lastly, a few representative batch and column experiments were also performed with S-nZVI, for direct comparison of reductive capacity to GR_SO4_. Moreover, in addition to the application of GR as engineered reactant, it is worth emphasizing the potential of naturally occurring GR materials for Cr(VI) removal in contaminated subsurface environments.

## Materials and methods

All solutions were prepared by dissolving reagent grade chemicals in deionized Milli-Q water (18.2 MΩ cm). GR _SO4_ and S-nZVI synthesis and all batch and column experiments were performed inside an anaerobic glovebox (Jacomex P[Box] Compact Glove Box, Dagneux, France) filled with an Ar atmosphere.

### GR_SO4_ synthesis

Sulphate green rust, GR_SO4_ (Fe(II)_4_Fe(III)_2_(OH)_12_SO_4_·8H_2_O) [40] was synthesized following the co-precipitation method used by Thomas et al. [23] (details given in Additional file 1: Text S1). The successful synthesis of GR_SO4_ was verified by X-Ray Diffraction (XRD) analysis performed with a Bruker D8 Advance X-ray diffractometer (Cu Kα) (Additional file 1: Figure S1). To determine the total and dissolved iron concentration in GR suspensions, acid digests of the GR suspension and its supernatant (by filtering through 0.2 μm syringe filter) were prepared and then analysed via Inductively Coupled Plasma Optical Emission Spectrometry (ICP-OES; Varian 715ES). This was then used to determine the concentration of total Fe(II) ([Fe(II)_tot_]) and Fe(II) in the solid ([Fe(II)_s_], assuming a Fe(II):Fe(III) ratio of 2 for GR_SO4_ (Additional file 1: Text S2). The dissolved Fe(II) concentration, [Fe(II)_d_], was approximated by the total dissolved Fe content, because the solubility of Fe(III) is very low at circum-neutral pH [43].

### GR_SO4_ batch studies

The reduction capacity and kinetics of Cr(VI) by GR_SO4_ was first studied in batch reactors with quartz sand for later comparison to flow experiments in sand columns. For this, the GR_SO4_ slurry (1 ml of 72.4 mM Fe(II)_tot_, pH 7) was mixed with 23 g quartz sand (size range: 0.1 - 0.3 mm, density: 2.66 g/m^3^, purchased in acid washed, dry state from Chemsolute Th. Geyer) and 5 ml MilliQ water inside 50 ml centrifuge tubes, and then spiked with 30 ml of separately prepared Cr(VI) stock solutions, produced by dissolving defined quantities of K_2_Cr_2_O_7_ in Milli-Q water, to yield final Cr(VI) contents of 7.5, 15.1 and 30.0 µmol, and Cr(VI)/Fe(II)_tot_ molar ratios of 0.1, 0.21 and 0.41. Note that at Cr(VI)/Fe(II)_tot_ ratios < 0.33, Fe(II)_tot_ will be in excess, i.e., all Cr(VI) can theoretically be reduced to Cr(III) by oxidation of Fe(II)_tot_ to Fe(III), while at ratios > 0.33, Cr(VI) will be in excess (i.e., there are insufficient GR_SO4_ reducing equivalents for full Cr(VI) reduction). The prepared batch reactions were shaken on an orbital shaker at 300 rpm at room temperature (~ 25 °C) for up to 24 h. To assess the decrease in Cr(VI) with time, two tubes were removed at regular sampling times (5, 10, 15, 30, 45 and 60 min) centrifuged and the supernatant filtered (0.2 µm syringe filter, Chromafil, Carl Roth Germany) for analysis of dissolved Cr(VI) using the 1,5-diphenylcarbazide colorimetric method (details given in the Additional file 1: Text S3). The solution pH was also monitored. A control experiment with sand and Cr(VI) only (no added GR_SO4_) showed that Cr(VI) sorption to grain surfaces or the sample tube was negligible (Additional file 1: Figure S3), thus any monitored decrease in Cr(VI) with time is due to removal by the added GR_SO4_.

Lastly, a sand-free batch experiment with only GR_SO4_ and Cr(VI) at pH 7 (Cr(VI)/Fe(II)_tot_ = 0.21) was set up to get sufficient material for XRD analysis of final GR_SO4_ oxidation products. Here, solids were retrieved after 24 h using centrifugation and prepared for XRD as described in Additional file 1: Text S1.

### GR_SO4_ column studies

Column experiments (diameter × length = 1.4 cm × 11 cm) were carried out to investigate the effect of initial Cr(VI) concentration (0.125–1 mM), flow rate (0.25, 1 and 3 ml/min) and solution pH (4.5, 7.0 and 9.5; adjusted by adding 1 M HCl or 1 M NaOH) on Cr(VI) removal by GR_SO4_ in porous media. These Cr(VI) concentrations are representative of conditions found at Cr(VI) contaminated sites. For example, Cr(VI) concentrations in a waste plume at the Hanford site, Washington (USA) range from 0.090 to 0.96 mM [44, 45], and reached around 0.5 mM in a contaminated groundwater plume at the U.S. Coast Guard Support Center, Elizabeth City, North Carolina [46]. Note that the chosen flow rates yield pore water velocities (calculated from the breakthrough curves of the tracer test, Eq. 1) that are generally higher than groundwater velocities in field PRBs (up to 2.2 m/day) [47]. However, such rates are technically hard to achieve in the laboratory. Also, if these materials are used for treatment either via injection or by installing a funnel-and-gate PRB, then rates will be closer to what we tested here. PRBs with a funnel-and-gate configuration consist of two impermeable walls that direct the contaminated groundwater towards the reactive area (gate) [48]. In this scenario, the groundwater passing through the gate will have a much higher velocity than the natural flow velocity [49].

**Fig. 1:**
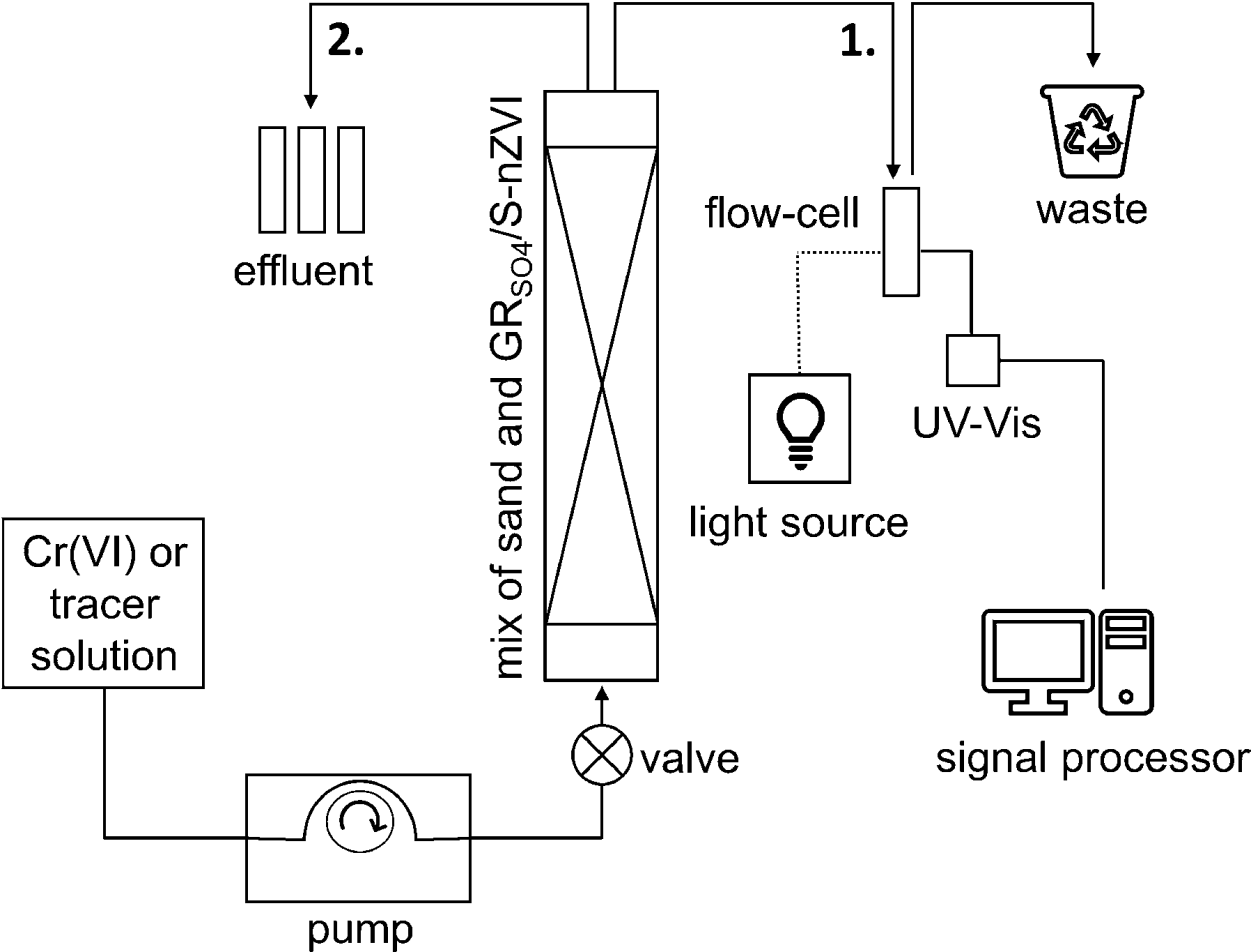
Experimental set-up used for measuring breakthrough curves under strictly anoxic conditions (1.) on-line by solution absorbance using a UV–Vis flow through cell connected to an UV–Vis spectrophotometer; (2.) manual collection to determine Cr(VI) via 1,5-diphenylcarbazide colorimetric method, and Cr_tot_ and Fe_tot_ via ICP-OES

The schematic diagram of the column set-up used to study Cr(VI) removal by GR_SO4_ is shown in Fig. 1. The columns were wet-packed with a homogeneous GR_SO4_-sand slurry, prepared the same way as for batch experiments (except the addition of Cr(VI) solutions). The in- and out-let areas were amended with coarse sand (~ 80 mg, size range: 0.6–1.3 mm, Carl Roth Germany) to ensure plug flow and prevent loss of small sand grains. To determine the hydrodynamic properties of the column (i.e., dispersivity) and the corresponding linear flow velocities [47, 50] (“Fitting breakthrough curves” section), a non-reactive tracer solution (0.4 M NaNO_3_) was injected into one column containing quartz sand only. We assume that the presence of the reactant (GR_SO4_) did not alter the hydrodynamic properties within the column owing to the very low reactant/sand ratio (about 1/2500). To avoid the formation of trapped air bubbles within the column, the column was constantly tapped during wet packing. The columns were oriented vertically with upward flow to avoid channelling due to gravity. Before any injection, approximately 5 pore volumes of deoxygenated Milli-Q water were pumped through the column (Ismatec IPC peristaltic pump) to displace any trapped gas bubbles and to obtain steady-state flow conditions. For all Cr(VI) experiments, chromate solutions were injected until full breakthrough was observed. As a control experiment to measure any potential Cr(VI) sorption to sand grains and/or the column walls, one column was packed with quartz sand only (no added GR_SO4_) and then flushed with a 0.5 mM Cr(VI) solution, using a flow rate of 1 ml/min.

The chromium and nitrate concentrations in the outlet were monitored in situ by solution absorbance using an UV–Vis flow through cell (2 mm pathlength, Hellma, Germany) connected to an UV–Vis spectrophotometer (Ocean Optics) (details in Additional file 1: Text S3, Figure S4). Periodically, samples were also manually collected to measure total Cr, Cr(VI) and total Fe concentrations to check for any mobile Cr(III) and any released Fe. Total Cr and Fe were determined by ICP-OES, while Cr(VI) concentrations were determined using the 1,5-diphenylcarbazide colorimetric method (Environmental Protection Agency colorimetric method, EPA Method 7196A) (details provided in the Additional file 1: Text S3). All experimental conditions were tested in duplicate at room temperature (~ 25 °C), with each replicate column experiment conducted on a different day and using a different reactant synthesis batch. This ensured that the observed differences accounted for slight variations in sand column texture and GR synthesis and confirmed reproducibility. For the graphs of the breakthrough curves, the mean values of these duplicates were plotted.

### S-nZVI batch and column experiments

Batch (with/without sand) and column experiments were set-up identically as for GR_SO4_ experiments and monitored using the same sampling procedures outlined in “GR_SO4_ batch studies” and “GR_SO4_ column studies” sections. Sulphidized nanoscale ZVI (S-nZVI) was synthesized following the procedure reported by Mangayayam et al. [51] (detailed description in Additional file 1: Text S1). Following S-nZVI synthesis and drying under vacuum, the S-nZVI slurry (for mixing with the sand) was prepared as follows: approximately 43.5 mg dry S-nZVI were weighed into a glass vial, amended with 100 ml MilliQ water, then crimped sealed for sonication outside the anaerobic chamber. After, 6 ml of this suspension were mixed with the sand for batch/column experiments. As for GR_SO4_ experiments, the S-nZVI content was kept constant between experiments. We assumed that the Fe^0^ core of S-nZVI represents around 73% of the total volume, hence we approximated the amount of Fe^0^ in batch and column reactors to be ~ 38 µmol (detailed calculations in Additional file 1: Text S2). In reactions with Cr(VI), Fe^0^ gets oxidised to Fe(II) and then Fe(III) [15, 52] thus each Fe^0^ atom donates 3 electrons. As such, S-nZVI batch and column experiments theoretically have ~ 2.4 times the amount of reducing equivalents compared to GR_SO4_ experiments.

### Theoretical background of breakthrough curve and data evaluation

The common procedure to evaluate overall column performance is by means of breakthrough curve (BTC) analysis. BTCs are constructed by normalizing effluent concentration profiles (e.g., C versus time or eluted pore volume, PV) with inlet concentration (C_0_) to yield plots of C/C_0_ versus time or PV.

#### Fitting breakthrough curves

The PV (i.e., total porosity) of the column was determined by assessing the mass loss upon drying of the water saturated sand column. The values of the pore water velocity (*v*) and longitudinal dispersion coefficients (*D*) can be calculated from the breakthrough measured for the tracer and following equations [50]:

1 $$v = \frac{L}{{t_{0.5} }}$$

2 $$D = \frac{{v^{2} }}{{8t_{0.5} }}\left( {t_{0.84} - t_{0.16} } \right)^{2}$$

where L is the length of the column, and t_0.16_, t_0.5_ and t_0.84_ correspond to C/C_0_ breakthroughs of 0.16, 0.5 and 0.84 of the non-reactive tracer.

#### Cr(VI) removal capacity

A typical breakthrough curve presents an “S” profile with its shape and steepness controlled by the removal efficiency of the reactant present in the porous medium with respect to the inlet concentration of the solute and the flow rate [53]. The breakthrough point is fixed arbitrary at values very close to zero [54], while the exhaustion point is usually fixed at a C value between 90 and 95% of *C*_*0*_. In this study, we decided to fix the breakthrough point at 5% of *C*_*0*_ and we assumed GR_SO4_ and S-nZVI to be exhausted when the effluent Cr(VI) concentration reaches 90% of *C*_*0*_.

The total injected volume, *V*_*inj*_ (ml), and total injected Cr(VI) mass (q_*total*_, µmol and mg) are calculated as follows [55]:

3 $$V_{inj} = Q t_{0.9}$$

4 $$q_{total} = \frac{{C_{0} V_{inj} }}{1000}$$

where *Q* is the volumetric flow rate (ml/min) and *t*_*0.9*_ is the time at *C/C*_*0*_= 0.9.

The quantity of Cr(VI) immobilized within the column (*Cr(VI)*_*q*_; µmol and mg) can be calculated by integrating the area *(A)* beneath the breakthrough curve obtained by plotting *C*_*0*_ − *C* as function of time within the limits of *t*_*0*_ and *t*_*0.9*_ as follows:

5 $$Cr\left( {VI} \right)_{q} = \frac{Q A}{1000} = \frac{Q}{1000}\mathop \smallint \limits_{t0}^{{t = t_{0.9} }} (C_{0} - C)dt$$

where *t*_*0*_ marks the point where 1 PV of Cr(VI) solution has passed through the column (and after subtracting the delay time due to the length of the inlet tubes).

The absolute *Cr(VI) removal* (mg/g) was calculated based on the amount of reductant, i.e., GR (9.1 × 10^−3^ g) and S-nZVI (2.6 × 10^−3^ g), added to batch and column experiment (details of calculations in Additional file 1: Text S2):

6 $$Cr\left( {VI} \right) removal = \frac{{Cr\left( {VI} \right)_{q} }}{reductant\, mass }$$

The *Cr(VI) removal efficiency (%)* was calculated relative to the amount of available reducing equivalents *(RE)* by the added reductants, i.e., GR_SO4_ (= [Fe(II)_s_]) and S-nZVI (= 3 × [Fe^0^]_s_):

7 $$Cr\left( {VI} \right) removal\, efficiency \left( \% \right) = \frac{{Cr\left( {VI} \right)_{q} }}{RE}$$

*RE* factor reflects the number of electrons needed to reduce Cr(VI) to Cr(III), which is equal to 1 in the case of GR_SO4_ (from Fe(II) to Fe(III)) and equal to 3 in the case of S-nZVI (from Fe^0^ to Fe(III)).

## Results and discussion

### GR_SO4_ batch studies

**Fig. 2:**
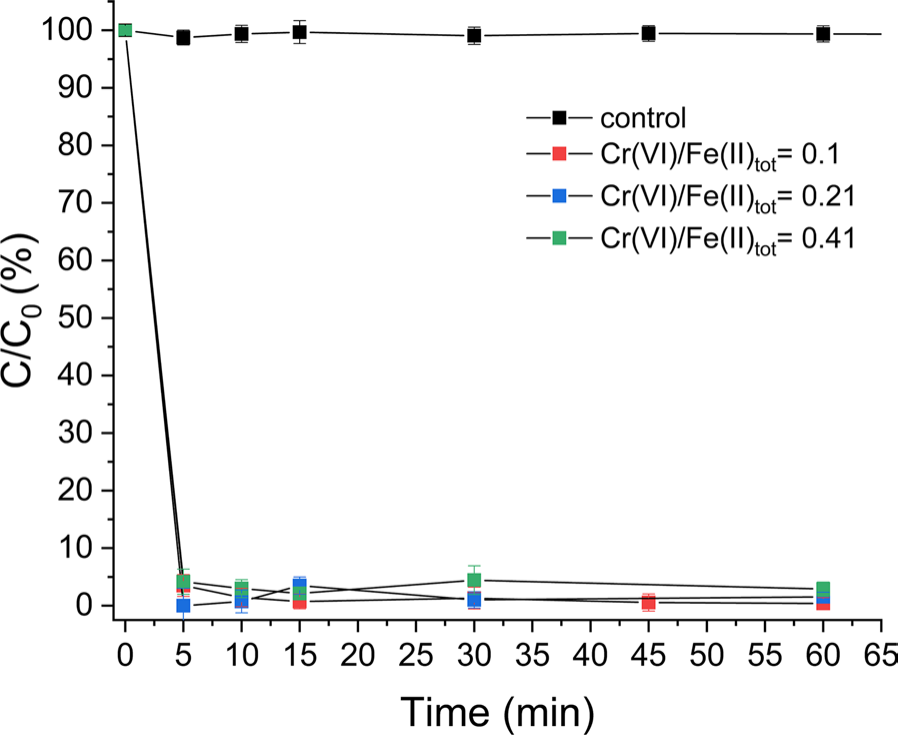
Cr(VI) immobilization efficiency by GR_SO4_ in batch sand experiments at variable Cr(VI)/Fe(II) ratio and constant green rust concentration. Control reaction with sand only (no added green rust) is also shown

The removal of Cr(VI) by GR_SO4_ in anoxic batch sand experiments is shown in Fig. 2. Under all tested Cr(VI) concentrations, removal rates were very fast with about > 96% of the initially added Cr(VI) removed already after 5 min, with little more Cr(VI) immobilised thereafter. Concomitantly, the pH decreased from 7 to 5 ± 0.25. This pH decrease can be explained by the reduction of Cr(VI) by the aqueous Fe(II) present in the added GR slurry (about 35.5% of Fe(II)_tot_), followed by the formation of Fe(III)-hydroxides as previously observed [3, 23]. Overall, these fast Cr(VI) removal rates match well with the removal rate observed in previous sand-free batch experiments [3, 23], indicating that the sand matrix had little effect on Cr(VI) immobilisation by GR_SO4_. XRD analyses of reaction products in the sand-free experiment after 24 h showed that most of the initial GR_SO4_ phase oxidised to goethite (α-FeOOH) (Additional file 1: Figure S2a). Note that these reactions were performed under strict anoxic conditions, thus the observed oxidation of GR_SO4_ is consistent with Cr(VI) removal by reduction to Cr(III) (Additional file 1: Figure S7). The fact that some GR remained after 24 h reaction is because there was an excess in Fe(II)_tot_, i.e., reducing equivalents, in this experiment. Overall, our observations are in agreement with previous GR_SO4_-Cr(VI) batch reactivity studies where Cr(III)-bearing goethite was identified as the primary oxidation product [23]. It is likely that some of the Cr(III) precipitated as amorphous Cr, Fe-phase, as observed in some studies with X-ray absorption spectroscopy [19], but this could not be resolved with the XRD used here.

In terms of Cr(VI) removal efficiencies relative to the added GR_SO4_ reducing equivalents, at the two lower tested Cr(VI) concentrations, where Fe(II)_tot_ was in excess [i.e., more reducing equivalents present than needed; Cr(VI)/Fe(II)_tot_ < 0.33], complete Cr(VI) removal was achieved within less than an hour of reaction. However, at the highest Cr(VI) concentration, where Cr(VI) was in excess [i.e., Cr(VI)/Fe(II)_tot_ > 0.33], 97% of the added Cr(VI) was immobilized. While incomplete removal was indeed expected for that experiment, because of insufficient reducing equivalents, it was actually 15% higher than the expected immobilization capacity based on reduction only. This may be due to additional Cr(VI) removal by Cr(VI) adsorption onto the newly formed goethite particles, which is favoured at the neutral pH studied here (i.e., below goethite zero point of charge of 9.1), as observed previously [56–58].

### GR_SO4_ column studies

The breakthrough curves for the tracer (NaNO_3_) as a function of differing injection flow rate (0.25, 1 and 3 ml/min) are shown in Additional file 1: Figure S5a. Under all conditions, the nitrate recovery was 100% demonstrating that it did not adsorb to the quartz sand grains in the column, and that collisions between nitrate and sand grains were elastic. From these data, the linear flow velocity (*v*) and longitudinal dispersion coefficient (*D*) were calculated (Eqs. 2 and 3, “Fitting breakthrough curves” section), yielding 8.72 × 10^−5^, 3.48 × 10^−4^ and 10.5 × 10^−4^ m/s (7.5, 30 and 90 m/day) and 1.81 × 10^−3^, 7.24 × 10^−3^ and 2.17 × 10^−2^ cm^2^/s, respectively, at the three different flow rates (i.e., 0.25, 1 and 3 ml/min). In turn, this meant that the residence times were 30, 7.5 and 2.5 min.

Similarly to the tracer experiments, control columns without added GR_SO4_ showed that all injected Cr(VI) (5 pore volumes of 0.5 mM) was retrieved at the outlet, i.e., no Cr(VI) adsorbed to quartz sand surfaces (Additional file 1: Figure S5b). This is also explained by the fact that both quartz sand and Cr(VI) species carry a net negative electric charge, thus repulsive electrostatic interactions will inhibit sorption processes between these compounds [59]. Moreover, analyses of the manually collected effluent samples of experimental columns by the colorimetric method (EPA Method 7196A) (total Cr(VI)) and ICP-OES (total Cr) matched the values measured using in situ spectrophotometry, reaffirming the suitability of the later method for experimental columns (Additional file 1: Figure S6). Also, we observed that the total Cr was equal to total Cr(VI) in the effluent of GR_SO4_ amended columns, which reaffirmed that the effluent contained no detectable Cr(III) species. Thus, any Cr(III) formed during reduction by GR_SO4_ must have become immobilised within the column (onto the surface of particles) by co-precipitation with the forming Fe(III) oxyhydroxides. It is worth noting that column experiments were not affected by significant pH changes during Cr(VI) injection, thus inlet and effluent pH values were identical.

Lastly, the aqueous Fe(II) that was initially present in the added GR slurry (~ 25 µmol) was mostly flushed out during the MilliQ water rinse (5 pore volumes) performed before each experiment (Additional file 1: Figure S8), thus the only reductant present within the columns was the added GR_SO4_ ([Fe(II)s] ~ 46.7 µmol).

#### Effect of initial inlet Cr(VI) concentration

**Fig. 3:**
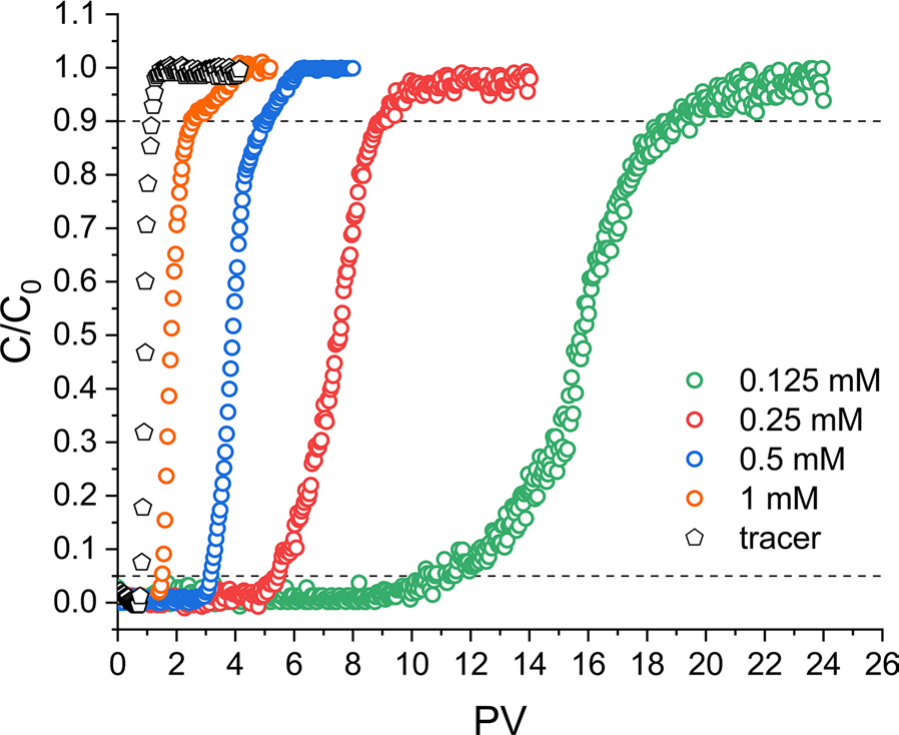
Cr(VI) breakthrough curves in GR_SO4_ amended packed sand columns as a function of different inlet Cr(VI) concentrations (C_0_ = 0.125, 0.25, 0.5 and 1 mM; flow rate = 1 ml/min; pH 7.0). The tracer test was performed with 0.4 M NaNO_3_. The dashed lines help to identify the breakthrough (C/C_0_ = 0.05) and the exhaustion (C/C_0_ = 0.9) points on each curve

Normalised experimental breakthrough curves obtained for GR_SO4_ amended sand columns as a function of inlet Cr(VI) concentrations, [Cr(VI)_0_], are shown in Fig. 3. By comparison to the tracer, it is clear that breakthrough of Cr(VI) was delayed in the presence of GR_SO4_ indicating that Cr(VI) was successfully immobilised by GR_SO4_.

Moreover, this delay in breakthrough became more pronounced with decreasing inlet Cr(VI) concentration, as shown by the breakthrough (C/C_0_ = 0.05) and exhaustion (C/C_0_ = 0.9) points, which increased from 1.5 to 11 PVs and from 2.5 to 19 PVs, respectively, for [Cr(VI)_0_] decreasing from 1 to 0.125 mM (Additional file 1: Table S1). This is expected because the lower the [Cr(VI)_0_], the larger the water volume that can be treated by the GR_SO4_ present, whose mass was identical in all experiments. In terms of absolute amounts of Cr(VI) removed, this value substantially decreased with increasing [Cr(VI)_0_], from about 76.6 mg to 29.3 mg Cr(VI) per g GR (Table 1). Along with the decreased Cr(VI) removal, we also observed that the breakthrough curves became significantly steeper with increasing [Cr(VI)_0_]. Overall, these observations strongly indicate that GR_SO4_ became more quickly exhausted at higher [Cr(VI)_0_].

In terms of Cr(VI) removal efficiency relative to the added GR_SO4_ reducing equivalents, our results show that not all added GR_SO4_ was oxidised in these reactions, and this was even more evident at higher [Cr(VI)_0_] (Table 1). The removal efficiency decreased from about 85% to 33% with an increase in [Cr(VI)_0_] from 0.125 to 1 mM. Such early GR_SO4_ exhaustion was not observed in batch sand experiments performed at the 3 different Cr(VI) concentrations. The occurrence of early GR_SO4_ exhaustion in columns can be explained by the constant Cr(VI) influx, which will keep reaction rates high on GR_SO4_ particle surfaces, which in turn is more likely to induce the formation of passivating reaction rims. Such passivating rims were shown by Skovbjerg et al. [19] and Williams and Scherer [21] in GR batch experiments with high reduction rates. This assumption is also supported by the fact that GR_SO4_ exhaustion occurs even faster if [Cr(VI)_0_] is higher. Moreover, some GR_SO4_ may not actually come in contact with the Cr(VI) solution, because of where the GR_SO4_ particles are situated in the columns (for example near to dead-ends and/or in static flow areas); however, that effect should have been similar amongst the different [Cr(VI)_0_] as the added GR_SO4_ mass and the flow rate were constant.

#### Effect of flow rate

**Fig. 4:**
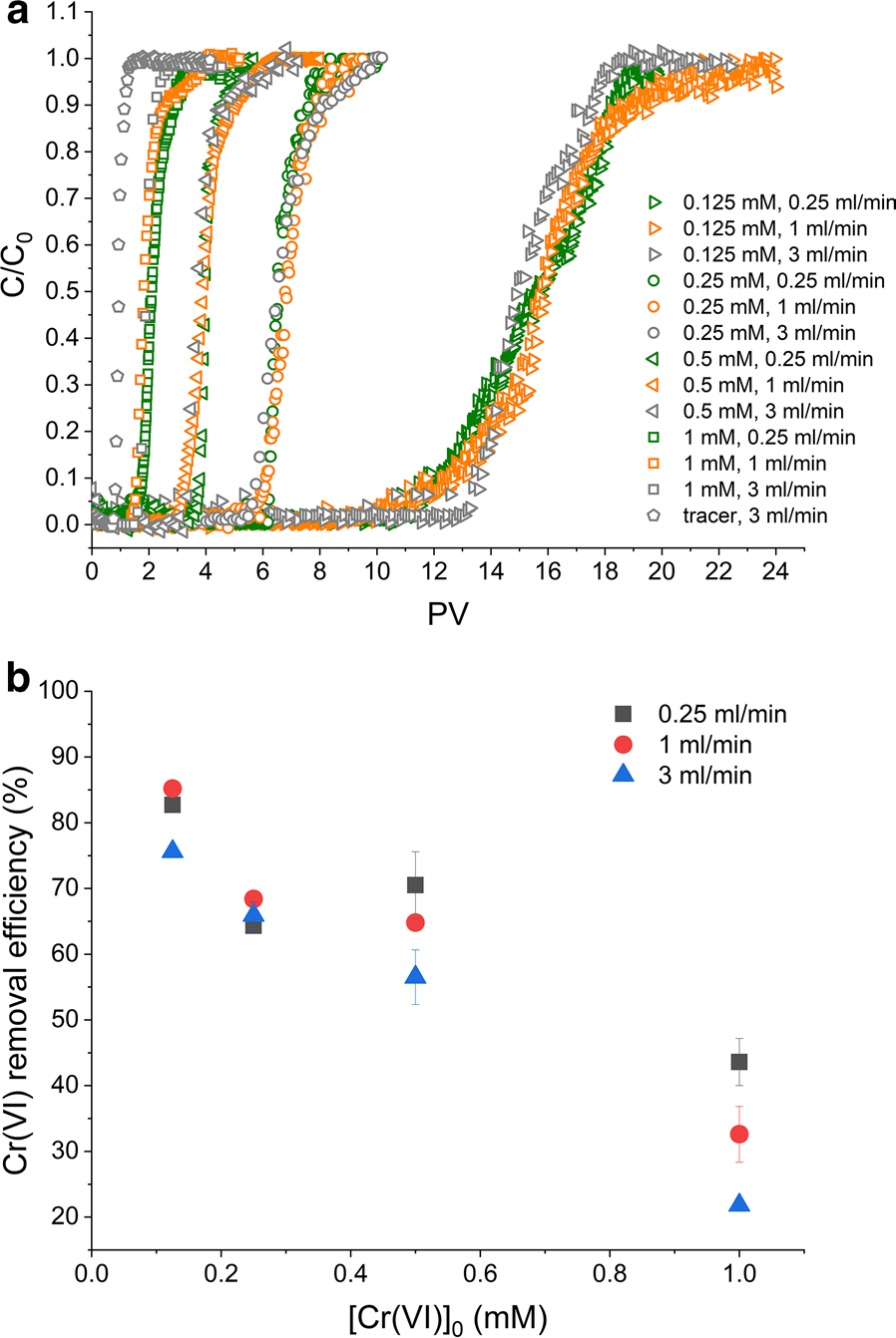
**a** Cr(VI) breakthrough curves and **b** calculated Cr(VI) removal efficiencies (%) in GR_SO4_ amended packed sand columns as function of different flow rate (0.25, 1 and 3 ml/min) at different inlet Cr(VI) concentrations (C_0_ = 0.125, 0.25, 0.5 and 1 mM; pH 7.0). BTCs (C/C_0_ vs PV) for the tracer test at different flow rates are identical

Normalised experimental breakthrough curves obtained for GR_SO4_ amended sand columns as a function of flow rate, i.e., 0.25, 1 and 3 ml/min (8.72 × 10^−3^, 3.48 × 10^−2^ and 10.5 × 10^−2^ cm/sec) for the different [Cr(VI)_0_] are shown in Fig. 4a. Overall, the above discussed trends with increasing [Cr(VI)_0_] do not greatly change with a change in flow rate: at all three tested flow rates, Cr(VI) removal (mg/g) and removal efficiencies (%) steadily decrease with increasing [Cr(VI)_0_] (Table 1). In terms of absolute values, it appears that these removal efficiencies are generally lower at higher flow rates, which is more clearly seen at higher [Cr(VI)_0_] (i.e., 0.5 and 1 mM, Fig. 4b). This decrease in removal efficiency with increasing flow rate is explained by the proportional decrease in contact time between GR_SO4_ particles and the Cr(VI) solution with increasing flow rate. Furthermore, with increasing flow rate, advection (i.e., flow through macropores) becomes more dominant, while flow close to pore surfaces decreases, and hence contact with immobilised GR_SO4_ is further reduced. At the lower [Cr(VI)_0_] (i.e., 0.125 and 0.25 mM), an increase in flow rate has less of an impact (Fig. 4b), because Cr(VI) reduction rates by GR are generally very high (as shown by batch sand experiments) and there seem to be sufficient GR_SO4_ particles in the flow path to react with the Cr(VI) solution.

**Table 1:** Amount of Cr(VI) immobilized within the column (Cr(VI)_q_, µmol), total Cr(VI) removal (mg/g) and Cr(VI) removal efficiency (%) calculated for different [Cr(VI)_0_], flow rates an inlet solution pH. Cr(VI) removal (mg/g) is the total amount of chromate immobilized (Cr(VI)_q_, mg) per grams of GR_SO4_ (9.1 mg) or S-nZVI (2.6 mg). Cr(VI) removal efficiency (%) is calculated assuming that GR_SO4_ and S-nZVI amended columns have 46.7 µmol (= 46.7 µmol Fe(II)) and 114 µmol (= 3 × 38 µmol Fe^0^) of reducing equivalents, respectively

Reactant type	[Cr(VI)_0_] (mM)	[Cr(VI)_0_] (mg/l)	pH	Q (ml/min)	Cr(VI)_q_ (μmol)	Cr(VI)_q_ (mg)	Cr(VI) removal (mg/g)	Cr(VI) removal efficiency (%)
GR_SO4_	0.125	6.5	7	0.25	12.9	0.67	74.4	82.7
			7	1	13.3	0.69	76.6	85.2
			7	3	11.8	0.61	68.0	75.6
GR_SO4_	0.25	13	7	0.25	10.0 ± 0.1	0.52 ± 0.1	57.8 ± 0.3	64.3 ± 0.6
			7	1	10.7 ± 0.1	0.55 ± 0.1	61.6 ± 0.3	68.4 ± 0.5
			7	3	10.3 ± 0.3	0.53 ± 0.3	59.2 ± 1.8	65.9 ± 2.9
GR_SO4_	0.5	26	7	0.25	11.0 ± 0.8	0.57 ± 0.8	63.4 ± 4.5	70.5 ± 7.1
			4.5	1	13.5 ± 0.3	0.70 ± 0.3	77.7 ± 1.6	86.4 ± 2.6
			7	1	10.1 ± 0.1	0.52 ± 0.1	58.2 ± 0.5	64.8 ± 0.8
			9.5	1	8.8 ± 0.7	0.46 ± 0.7	50.8 ± 4.2	56.5 ± 6.6
			7	3	8.7 ± 0.7	0.45 ± 0.7	50.1 ± 3.7	55.7 ± 5.9
GR_SO4_	1	52	7	0.25	6.8 ± 0.6	0.35 ± 0.6	39.2 ± 3.2	43.5 ± 5.1
			7	1	5.1 ± 0.7	0.26 ± 0.7	29.3 ± 3.8	32.6 ± 6.0
			7	3	3.4 ± 0.1	0.18 ± 0.1	19.6 ± 0.5	21.8 ± 0.7
S-nZVI	0.25	13	4.5	0.25	2.7 ± 0.1	0.14	53.4 ± 0.8	7.05 ± 0.9
			7	0.25	1.3 ± 0.1	0.07	26.2 ± 0.5	3.47 ± 0.8
			7	1	1.0 ± 0.1	0.05	20.7 ± 0.3	2.73 ± 0.5
			7	3	1.1 ± 0.1	0.06	22.1 ± 0.4	2.91 ± 0.7

#### Effect of pH

**Fig. 5:**
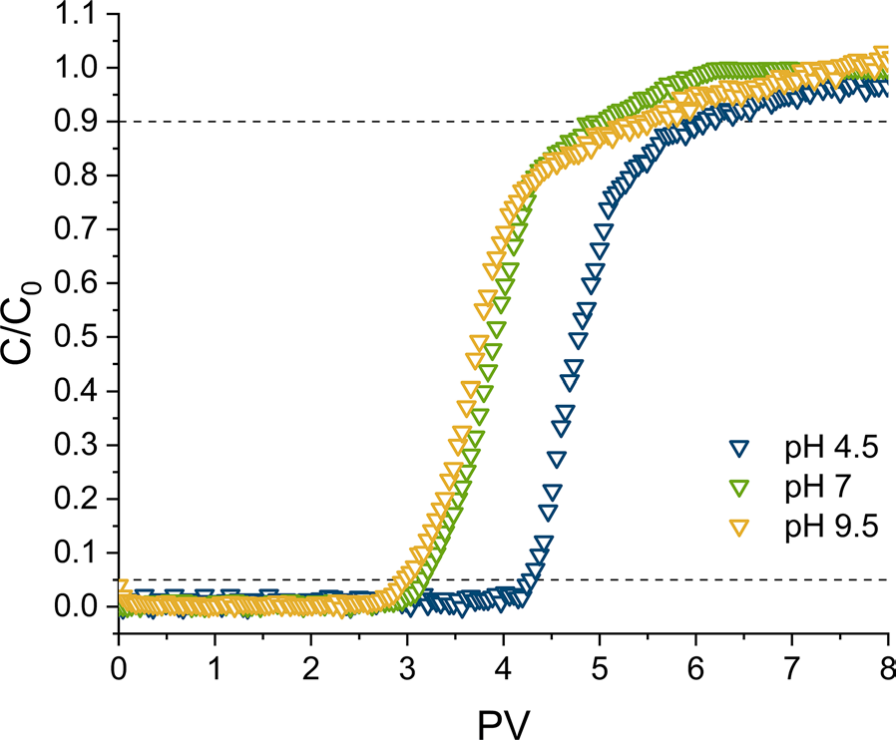
Cr(VI) breakthrough curves in GR_SO4_ amended packed sand columns as a function of inlet solution pH: 4.5, 7.0 and 9.5 ([(Cr(VI)_0_] = 0.5 mM, flow rate = 1 ml/min). The dashed lines help to identify the breakthrough (C/C_0_ = 0.05) and exhaustion (C/C_0_ = 0.9) points on each curve

Normalised experimental breakthrough curves obtained for GR_SO4_ amended sand columns as a function of inlet solution pH (4.5, 7.0 and 9.5), where [Cr(VI)_0_] and the flow rate were kept constant at 0.5 mM and 1 ml/min, respectively, are shown in Fig. 5. Note that even in the pH 4.5 treatment, Cr(VI) was still the main dissolved Cr species as determined by solution analyses.

The breakthrough at pH 7.0 and 9.5 are fairly similar, although calculated absolute Cr(VI) removal and removal efficiencies were a little lower at pH 9.5 compared to pH 7.0 (Table 1), but still within experimental error. In contrast, the breakthrough at pH 4.5 occurred substantially later (Fig. 5), meaning a greater amount of Cr(VI) could be immobilised at this lower inlet pH (79.3 mg/g) compared to results at pH 7.0 and 9.5 (~ 58 mg/g, Table 1). It is important to mention that GRs are fairly stable at pHs between 6.5 and 10 (also depending on interlayer anion and geochemical conditions), while they will dissolve and/or transform to other phases at pHs above or below this range [60, 61]. We argue that the enhanced Cr(VI) immobilisation at acidic pH is likely triggered by GR dissolution, and subsequent release of aqueous Fe(II) that, in combination with the remaining Fe(II)_s_, can readily react with the Cr(VI). The increased solubility of green rust and the dominance of aqueous Fe^2+^ over green rust under acidic conditions have been demonstrated in previous studies [61–64]. Furthermore, this reduction might be enhanced by the remaining GR_SO4_, whose surface acts as catalyst [19]. Indeed, under these acidic conditions, it is also less likely that passivation rims form on GR particles [19], thus the reaction can proceed for longer. For comparison, Williams and Scherer [21] also observed an increase in Cr(VI) reduction rates with a decrease in pH from 9 to 5 in batch experiments, and argued that dissolution is likely responsible for this trend. It is worth noting that further processes such as (ad)sorption of Cr(VI) by GR oxidation products (e.g., goethite or ferrihydrite) might also be enhanced at acidic conditions and add to the observed immobilization, leading to a higher removal performance [57]. However, at acidic conditions the Cr(VI) removal is mainly attributable to the reductive precipitation of dissolved Cr(VI) by Fe(II) (Additional file 1: Figure S7). Under all three tested pH conditions, reduction of Cr(VI) to Cr(III) will therefore result in the formation of an insoluble Cr,Fe phase (see Sect. 3.1).

### Cr(VI) removal capacity by S-nZVI

**Fig. 6:**
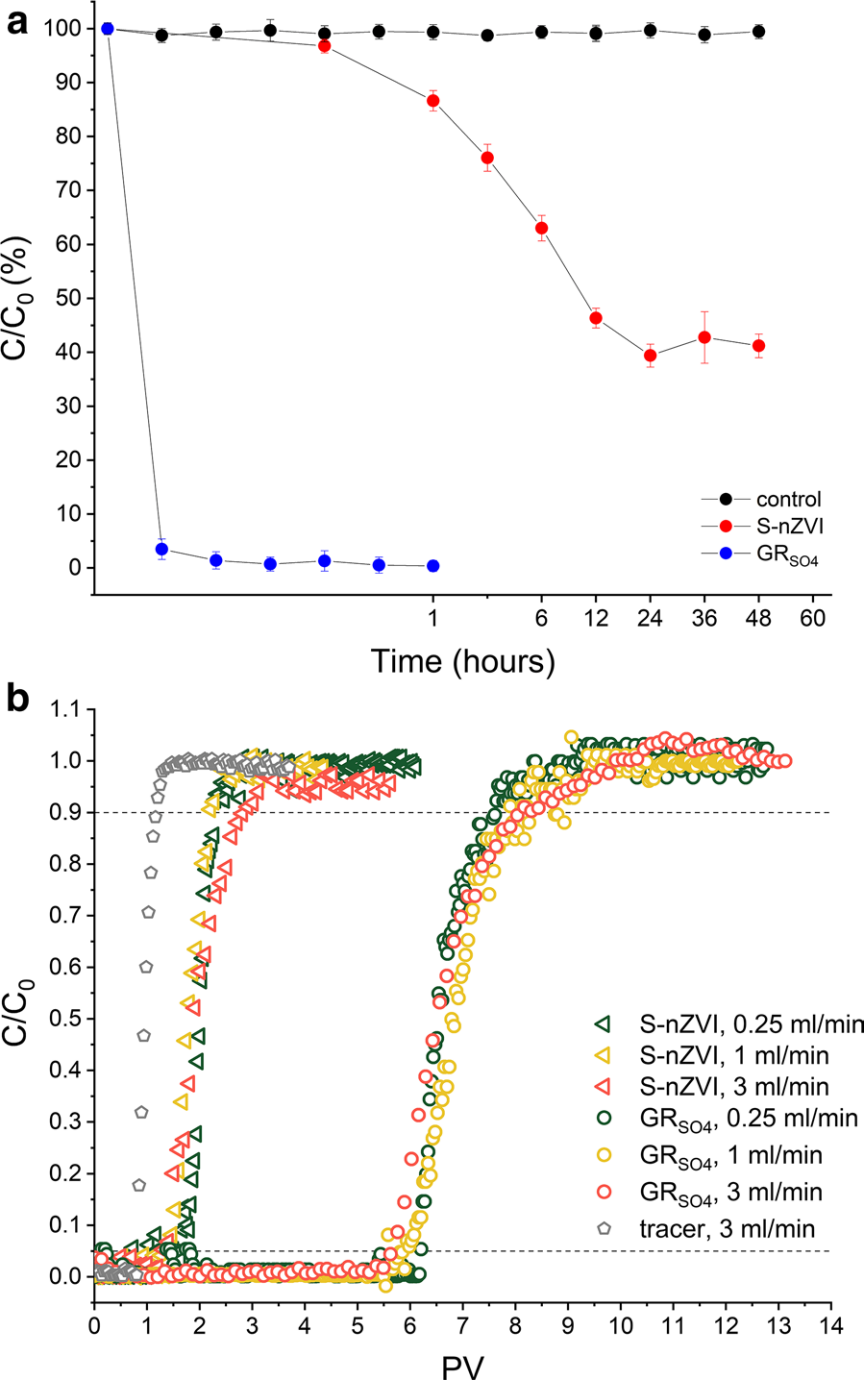
**a** Cr(VI) immobilization efficiency by S-nZVI and GR_SO4_ (0.25 mM [Cr(VI)_in_]). A control experiment with no added reducing agent is also shown. Each measurement represents one batch reaction (note that x-axis is not linear). **b** Cr(VI) breakthrough curves in S-nZVI (triangles) and GR_SO4_ (circles) amended sand columns as a function of flow rates: 0.25, 1 and 3 ml/min ([(Cr(VI)_0_] = 0.25 mM and pH 7.0). The black dotted lines mark the breakthrough (C/C_0_ = 0.05) and exhaustion (C/C_0_ = 0.9) points on each curve. BTCs (C/C_0_ vs PV) for the tracer test at different flow rates are identical

For comparison to GR_SO4_, a representative set of batch and column experiments were performed with sulphidised nZVI (S-nZVI). Figure 6a shows that the removal of Cr(VI) by S-nZVI in batch sand experiments is significantly slower compared to GR_SO4_, despite the 1.5× higher amount of reducing equivalents in S-nZVI reactions compared to GR_SO4_. Moreover, Cr(VI) removal by S-nZVI slows down considerably after 12 h, and little change is observed thereafter (Fig. 6a), yielding a maximum Cr(VI) removal of ~ 60% within 48 h. In contrast, in GR_SO4_ reactions nearly complete removal was observed within 30 min, indicating not only a higher removal efficiency but also higher reaction kinetics for GR_SO4_ compared to S-nZVI.

The lower reactivity of S-nZVI with Cr(VI) compared to GR_SO4_ may be explained by S-nZVI particles forming larger aggregates due to magnetic attraction [65], thus available reactive surface area may be much lower than expected. Also, it is argued that Cr(VI) can be reduced on GR surfaces and in its interlayer, providing abundant reactive sites for reduction [20, 22, 42]. Additionally, the observation that Cr(VI) removal was incomplete in S-nZVI batch reactions despite sufficient S-nZVI reducing equivalents, suggests that S-nZVI surfaces became passivated by oxidised Fe phases. This is supported by XRD analysis of solids after 24 h reaction between S-nZVI and Cr(VI) in sand-free batch reactors that show the characteristic peaks of the initial S-nZVI phase, i.e., the Fe^0^ core and the FeS shell (Additional file 1: Figure S2b), but also characteristic peaks of oxidised Fe (oxyhydr)oxide phases, including lepidocrocite and magnetite. As for GR_SO4_ experiments, some amorphous Cr,Fe phases may have also formed in S-nZVI reactions, but could not be identified with XRD here. These oxidised Fe phases have lower zero point of charge (lepidocrocite: 7.1 [66] and magnetite: 6.5 [67]) compared to goethite (which is the oxidation product in GR_SO4_ reactions), thus Cr(VI) removal by adsorption is also less favoured in S-nZVI reactions.

In terms of S-nZVI reduction performance in columns, Fig. 6b shows the normalised experimental breakthrough curves obtained for S-nZVI amended sand columns (~ 38 µmol of Fe^0^) as a function of flow rate, i.e., 0.25, 1 and 3 ml/min for [Cr(VI)_0_] = 0.25 mM. For comparison, breakthrough curves obtained in GR_SO4_ amended sand columns at the same flow rates and [Cr(VI)_0_] conditions are also shown. Similar to the GR_SO4_ column results, flow rate had little effect on absolute Cr(VI) removal and removal efficiencies in S-nZVI columns, yielding 26.2, 20.7 and 22.1 mg/g and 3.47%, 2.73% and 2.91% at 0.25, 1 and 3 ml/min, respectively, while lower pH conditions enhanced Cr(VI) removal (53.4 mg/g and 7.05%) (Additional file 1: Figure S9). The breakthrough (C/C_0_ = 0.05) and exhaustion (C/C_0_ = 0.9) points under those conditions are listed in Additional file 1: Table S1. Our findings are in good agreement with Gong et al. [15], who showed that immobilization of Cr(VI) by S-nZVI is strongly dependent on pH conditions, with higher performance at lower pH. Similar to GR_SO4_ observations, Cr(VI) removal in S-nZVI amended columns is significantly lower than in batch sand experiments (Table 1). However, the difference is much more pronounced in the S-nZVI system because of its much lower Cr(VI) reduction rate. This in turn suggests that a higher removal efficiency could potentially be achieved in the column, if the contact time is increased, i.e., flow rate is further decreased. However, surface passivation as observed in batch reactions is still a major concern in the application of S-nZVI, so it is doubtful that efficiencies would increase that much in the columns even if flow rates are considerably decreased.

## Conclusion and implications

This study demonstrated the high effectiveness of GR_SO4_ to immobilise mobile Cr(VI) inside porous sand media. Batch sand studies confirmed similar fast reduction of Cr(VI) by GR_SO4_ as observed for batch studies in aqueous media (i.e., where no sand was added), with > 95% Cr(V) removed within only 10 min. In comparison, Cr(VI) removal efficiencies in sand columns under dynamic flow conditions were substantially lower than in batch studies, particularly at higher inlet Cr(VI) concentrations. This is likely explained by the constant influx of Cr(VI) solution which keeps reaction rates high on GR particle surfaces likely promoting the formation of passivating rims on GR surfaces, as observed before in batch experiments with high initial Cr(VI) concentrations. Furthermore, lower Cr(VI) removal efficiencies were observed at higher flow rates and alkaline pH conditions (compared to acidic). For comparison, similar batch and column studies were also performed with S-nZVI, an alternative reductant material. These results clearly showed that Cr(VI) reduction and immobilisation by GR_SO4_ is substantially faster and yields 2.5 times higher efficiencies compared to S-nZVI, meaning GR_SO4_ performs substantially better under the tested flow conditions.

Overall, these results demonstrate the high potential for use of GR_SO4_ in Cr(VI) remediation applications, given the presence of sand matrices, dynamic flow conditions and changing pH conditions, we still observe high immobilisation yields that are considerable higher than what is observed for alternative reductants such as S-nZVI. Moreover, the observed trends suggest that at much lower flow rates, Cr(VI) removal efficiencies by GR_SO4_ would likely be much higher. Thus, at flow rates closer to average groundwater flow (e.g., 6–220 cm/day), GR_SO4_ might achieve 100% removal efficiency because of higher contact time with Cr(VI).

Obviously, any Cr(VI) contaminated site has its own characteristics depending on the hydrogeological and geochemical properties of the contaminated subsurface (sand, gravel or unconsolidated sediments, presence of organic matter/biology, permeability, aerobic vs anaerobic), and remediation with GR_SO4_ as well as S-nZVI may not necessarily suit all sites. Overall, with this study we show that, under specific conditions, both GR_SO4_ and S-nZVI can successfully reduce and immobilise Cr(VI) in porous media. Therefore, these materials certainly warrant further study on how to apply them at larger scale.

## Supporting Information

Detailed description of the GR_SO4_ and S-nZVI synthesis, mineral characterization of oxidation products, manual and on-line measurements of effluent aqueous samples, as well as breakthrough analysis procedures, can be found in the attached Additional file 1.

## Supplementary information

**Additional file 1: Text S1.** Synthesis and characterization of GR_SO4_ and S-nZVI. **Figure S1.** XRD patterns (Cu-Kα) of freshly synthesized and glycerol-coated a) GR_SO4_ (PDF# 13-0092) and (b) S-nZVI (Fe^0^ PDF# 06-0696 and FeS PDF# 89-6268). **Figure S2.** XRD patterns (Cu-Kα) of oxidation products after reaction with Cr(VI) solution (pH 7) of a) GR_SO4_: goethite (α-FeOOH, PDF# 29-0713) and GR_SO4_, and (b) S-nZVI: Fe^0^, FeS, lepidocrocite (PDF# 44-1415) and magnetite (PDF# 19-0629). **Text S2.** Calculation of the iron content, mass and volume of GR_SO4_ and S-nZVI. **Figure S3.** Control batch experiment with sand and Cr(VI) only (no added GR_SO4_ or S-nZVI) showed that Cr(VI) sorption to grain surfaces was negligible over 48 h (note that x-axis is not linear). **Text S3.** NaNO_3_, Cr(VI) and total Cr measurements. **Figure S4.** (a) Effect of Cr(VI) solution pH (4.5, 7.0 and 9.5) on UV–Vis absorption spectra; (b) UV–Vis spectra of Cr(VI) solutions of varying concentration (0.125 and 2 mM; at pH 7) used to make the calibration curves shown in (c) and (d) where adsorption readings were taken at 274.3 (R2 = 0.999) and 371.3 nm (R2 = 0.998), respectively. **Figure S5.** (a) BTCs (as a function of time) obtained by injecting 0.4 M NaNO_3_ at 0.25, 1 and 3 ml/min. (b) Comparison of BTCs (as a function of pore volume) obtained by injecting 0.5 mM Cr(VI) solution (control) at 0.25, 1 and 3 ml/min and 0.4 M NaNO_3_ solution (tracer) at 1 ml/min. All BTCs show no delay in breakthrough, i.e., are typical of non-reactive solutes. **Figure S6.** Comparison of BTCs obtained by measuring effluent Cr(VI) concentrations using the on-line UV–Vis set-up (black symbols) and by determining total Cr concentrations via ICP-OES in manually collected samples (red symbols). The test conditions were identical with [Cr(VI)_0_] = 0.5 mM, pH = 7 and flow rate = 1 ml/min. The fact that the two BTCs overlap demonstrates that Cr(VI) is the only Cr species detected in the effluent. Thus, any Cr(III) forming due to reduction is immobilised within the sand column. **Figure S7.** Eh–pH diagram for chromium based on experimental chromium concentration (10^−3^ M). Dashed line is based on lower concentrations (10^−6^ M). Calculations were made using PHREEQC (USGS). **Figure S8.** Prior to each Cr(VI) injection, columns were pre-flushed with 5 PVs of MilliQ water to remove aqueous Fe^2+^ present in the GR slurry that was mixed with the sand. Manually collected samples were analysed via ICP-OES to determine loss in total Fe as a function of flushed MilliQ pore volumes, which is depicted here. The plotted data show average values of 6 columns (3 columns run at 1 ml/min and 3 run at 3 ml/min). **Figure S9.** Cr(VI) breakthrough curves in S-nZVI amended packed sand columns as a function of inlet solution pH: 4.5 and 7.0 ([(Cr(VI)_0_] = 0.25 mM, flow rate = 0.25 ml/min). The dashed lines help to identify the breakthrough (C/C_0_ = 0.05) and exhaustion (C/C_0_ = 0.9) points on each curve. **Table S1.** Breakthrough (C/C_0_ = 0.05) and exhaustion (C/C_0_ = 0.9) points expressed in terms of pore volumes (PV) for GR_SO4_ and S-nZVI sand column experiments performed at different [Cr(VI)_0_], flow rates and solution pHs (data taken from Fig. 4a, 5 and 6b).

## Data Availability

The datasets are available from the corresponding author upon request.

## Abbreviations

GR_SO4_: Sulphate green rust
P&T: Pump-and-treat
nZVI: Nanoscale zerovalent iron
XRD: X-Ray Diffraction
ICP-OES: Inductively Coupled Plasma Optical Emission Spectrometry
BTC: Breakthrough curve
PV: Pore volume
