# Supplementary material for: Immobilization of Cr(VI) by sulphate green rust and sulphidized nanoscale zerovalent iron in sand media: batch and column studies
Source: Geochem Trans. 2020 Aug 14;21:8. doi: 10.1186/s12932-020-00073-9 (PMC7429723; doi:10.1186/s12932-020-00073-9)
Supplement: Supplementary file 1 — Additional file 1: Text S1. Synthesis and characterization of GRSO4 and S-nZVI. Figure S1. XRD patterns (Cu-Kα) of freshly synthesized and glycerol-coated a) GRSO4 (PDF# 13-0092) and (b) S-nZVI (Fe0 PDF# 06-0696 and FeS PDF# 89-6268). Figure S2. XRD patterns (Cu-Kα) of oxidation products after reaction with Cr(VI) solution (pH 7) of a) GRSO4: goethite (α-FeOOH, PDF# 29-0713) and GRSO4, and (b) S-nZVI: Fe0, FeS, lepidocrocite (PDF# 44-1415) and magnetite (PDF# 19-0629). Text S2. Calculation of the iron content, mass and volume of GRSO4 and S-nZVI. Figure S3. Control batch experiment with sand and Cr(VI) only (no added GRSO4 or S-nZVI) showed that Cr(VI) sorption to grain surfaces was negligible over 48 h (note that x-axis is not linear). Text S3. NaNO3, Cr(VI) and total Cr measurements. Figure S4. (a) Effect of Cr(VI) solution pH (4.5, 7.0 and 9.5) on UV–Vis absorption spectra; (b) UV–Vis spectra of Cr(VI) solutions of varying concentration (0.125 and 2 mM; at pH 7) used to make the calibration curves shown in (c) and (d) where adsorption readings were taken at 274.3 (R2 = 0.999) and 371.3 nm (R2 = 0.998), respectively. Figure S5. (a) BTCs (as a function of time) obtained by injecting 0.4 M NaNO3 at 0.25, 1 and 3 ml/min. (b) Comparison of BTCs (as a function of pore volume) obtained by injecting 0.5 mM Cr(VI) solution (control) at 0.25, 1 and 3 ml/min and 0.4 M NaNO3 solution (tracer) at 1 ml/min. All BTCs show no delay in breakthrough, i.e., are typical of non-reactive solutes. Figure S6. Comparison of BTCs obtained by measuring effluent Cr(VI) concentrations using the on-line UV–Vis set-up (black symbols) and by determining total Cr concentrations via ICP-OES in manually collected samples (red symbols). The test conditions were identical with [Cr(VI)0] = 0.5 mM, pH = 7 and flow rate = 1 ml/min. The fact that the two BTCs overlap demonstrates that Cr(VI) is the only Cr species detected in the effluent. Thus, any Cr(III) forming due to reduction is immobilised within [file 12932_2020_73_MOESM1_ESM.docx]

**Additional Information**

**Immobilization of Cr(VI) by sulphate green rust and sulphidized nanoscale zerovalent iron in sand media: batch and column studies**

Flavia Digiacomo^1,2*^, Dominique J. Tobler^3^, Thomas Held^4^, Thomas Neumann^5^

^1^ARCADIS Germany GmbH, Griesbachstraße 10, 76185, Karlsruhe, Germany

^2^Institute of Applied Geosciences, Karlsruhe Institute of Technology, Adenauerring 20b, Building 50.40, 76131 Karlsruhe, Germany

^3^Nano-Science Center, Department of Chemistry, University of Copenhagen, Universitetsparken 5, 2100 Copenhagen, Denmark

^4^ARCADIS Germany GmbH, Europaplatz 3, 64293 Darmstadt, Germany

^5^Department of Applied Geosciences, Technical University of Berlin, Ernst-Reuter-Platz 1, 10587 Berlin, Germany

* Corresponding author: flavia.digiacomo@kit.edu

This additional file contains: 10 pages, 9 figures and 1 table

**Additional details towards methods**

# Text S1: Synthesis and characterization of GR_SO4_ and S-nZVI

*Sulphate green rust, GR_SO4_* ((Fe(II)_4_Fe(III)_2_(OH)_12_SO_4•_8H_2_O), was synthesized following the co-precipitation method used by Thomas *et al*.[1] In short, 40 ml of a 33 mM Fe_2_(SO_4_)_3_ solution were slowly titrated (1 ml/min, Ismatec IPC peristaltic pump) to 20 ml 67 mM FeSO_4_·7H_2_O inside a polypropylene beaker under constant stirring (300 rpm) inside an argon glovebox. The solution pH was maintained at approximately 7.0 using dropwise addition of 1 M NaOH. Following titration, the green rust suspension was stirred for 24 hours and then stored at room temperature inside the glovebox until use (maximum of 5 days).

*Sulphdized nano zerovalent iron**, S-nZVI,* was synthesized in an argon glovebox as follows: first, nanoscale zerovalent iron was synthesised by titrating 71.6 mL of 0.25 M FeCl_2_•4H_2_O solution with 35.8 ml of 1 M NaBH_4_ at a rate of 5 mL min^-1^ using a peristaltic pump. The resulting solution was mechanically stirred for another 15 minutes before separating the formed nZVI particles using a strong magnet. The separated nZVI were resuspended in 120.4 mL of 0.2 M acetate buffer (pH 5.7) inside 160 mL serum bottle, crimp sealed and sonicated for 15 minutes. Thereafter, 4.2 mL of 1 M Na_2_S was added to the suspension, sonicated for another 5 minutes and then placed on an orbital shaker (180 rpm) for 3 hours. Afterwards, the particles were washed thrice with degassed 96% ethanol.

*X-Ray Diffraction (XRD) analyses*: The material suspensions were vacuum filtered (Whatman 0.2 µm nylon membrane filter) and amended with glycerol to prevent oxidation during XRD analysis [2–4]. XRD was performed using a Bruker D8 Diffractometer (Karlsruhe, Germany), with X-rays emitted from a Cu-Kα source (λ = 1.5418 Å). Data were collected at 2θ values between 2 and 72, with a step size of 0.02 and dwell time of 1 s per step (Fig. S1). XRD patterns were analysed with the EVA 100 software.

The oxidation products of GR_SO4_ and S-nZVI after 24-hour reaction with Cr(VI) were mounted on quartz slides inside the anoxic glovebox and examined via XRD. Data were collected at 2θ values between 2 and 72, with a step size of 0.02 and dwell time of 1 s per step (Fig. S2).


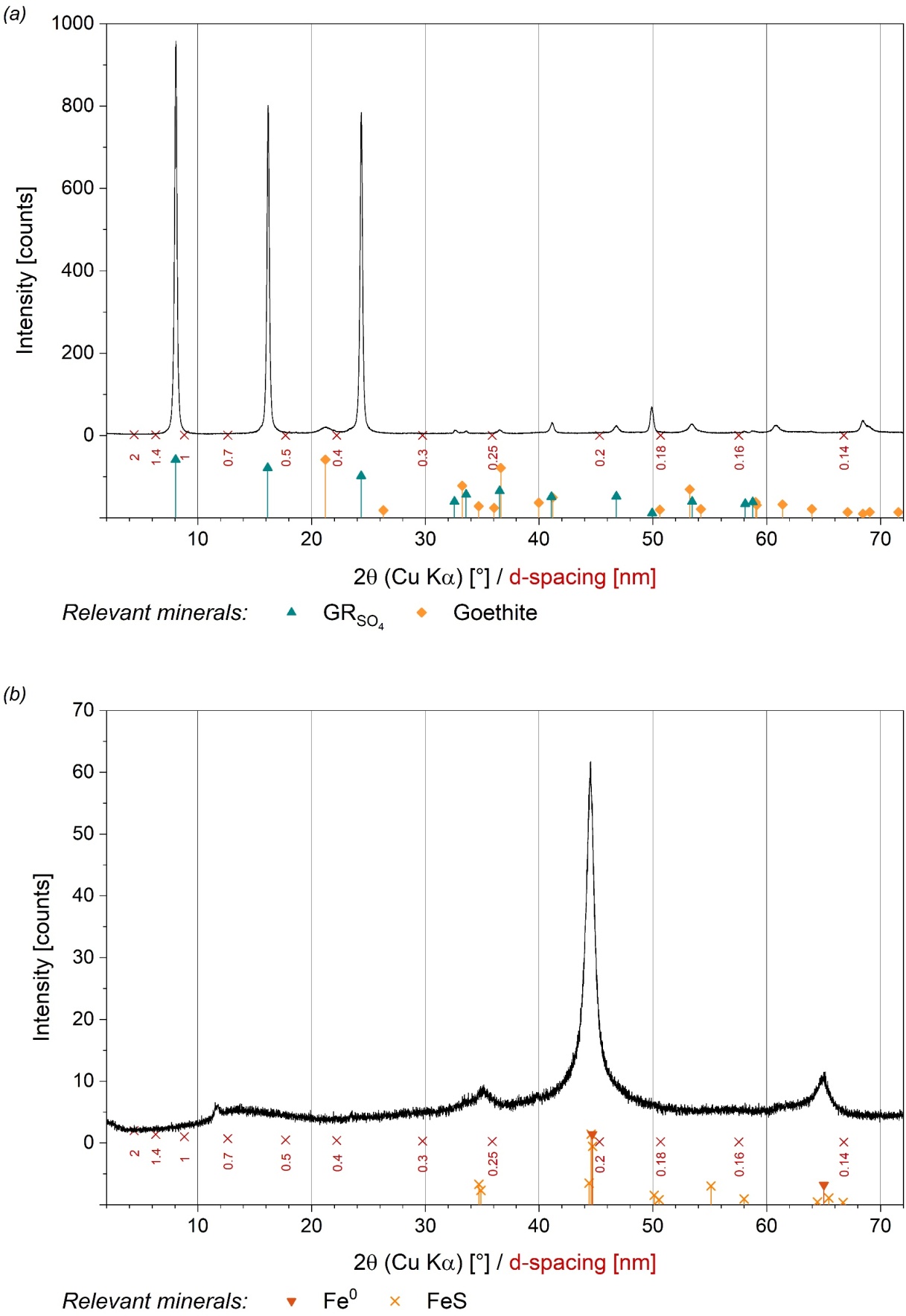


Figure S1: XRD patterns (Cu-Kα) of freshly synthesized and glycerol-coated a) GR_SO4_ (PDF# 13-0092) and (b) S-nZVI (Fe0 PDF# 06-0696 and FeS PDF# 89-6268).


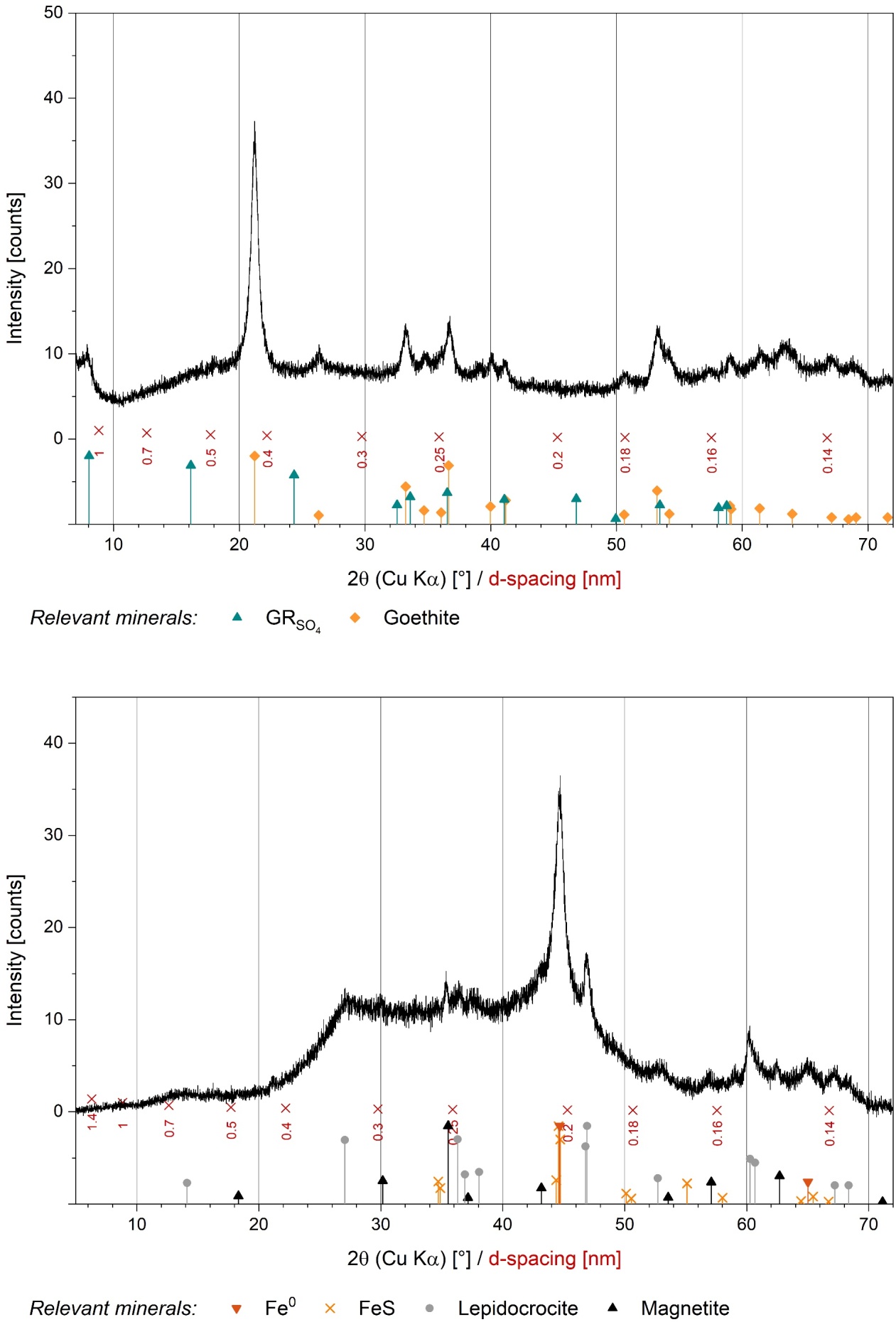


Figure S2 XRD patterns (Cu-Kα) of oxidation products after reaction with Cr(VI) solution (pH 7) of a) GR_SO4_: goethite (α-FeOOH, PDF# 29-0713) and GR_SO4_, and (b) S-nZVI: Fe^0^, FeS, lepidocrocite (PDF# 44-1415) and magnetite (PDF# 19-0629).

# Text S2: Calculation of the iron content, mass and volume of GR_SO4_ and S-nZVI

*Iron(II) content and GR_SO4_ mass:* To determine the total and dissolved iron content, [Fe_tot_] and [Fe_d_], of the GR suspension used for the batch and column experiments, we performed acid digestions of the GR suspension and its supernatant (obtained by filtering through 0.2 µm syringe filter). Samples were then analysed via Inductively Coupled Plasma Optical Emission Spectrometry (ICP-OES; Varian 715ES) at λ= 259.940 nm.

We measured:

$$\mathbf{[Fe}_{\mathbf{tot}}\mathbf{]=95.7}\mathbf{mmol}/\mathbf{l}$$

$\mathbf{[Fe}_{\mathbf{d}}\mathbf{]= 25.7}\mathbf{mmol}/\mathbf{l}$

Thus, the concentration of Fe in the solid, [Fe_s_] (e.g., GR particles), is:

$\mathbf{[Fe}_{\mathbf{s}}\mathbf{]=}\mathbf{[Fe}_{\mathbf{tot}}\mathbf{-}\mathbf{Fe}_{\mathbf{d}}\mathbf{]=70}\mathbf{mmol}/\mathbf{l}$ *Eqn. 1*

Next, we assume that (i) the Fe(II)/Fe(III) ratio in GR_SO4_ is 2 and (ii) the total dissolved Fe [Fe_d_] is mainly consisting of dissolved Fe(II) (the solubility of Fe(III) is very low at circum-neutral pH).[5] Based on this, the concentration of Fe(II) in the solid ([Fe(II)_s_]), and total Fe(II) in the GR_SO4_ suspension ([Fe(II)_tot_]) can be calculated as follows:

$\mathbf{[Fe(II)}_{\mathbf{s}}\mathbf{]=}\mathbf{[Fe}_{\mathbf{s}}\mathbf{]}\boldsymbol{\times}\frac{\mathbf{2}}{\mathbf{3}}\mathbf{=46.7}\mathbf{mmol}/\mathbf{l}$ *Eqn. 2*

$\mathbf{[Fe(II)}_{\mathbf{tot}}\mathbf{]=}\left( \mathbf{Fe}_{\mathbf{d}}\mathbf{+}\mathbf{Fe(II)}_{\mathbf{s}} \right)\mathbf{mmol}/\mathbf{l}\mathbf{=72.4}\mathbf{mmol}/\mathbf{l}$ *Eqn. 3*

Next, the mass of GR_SO4_, m(GR_SO4_)_susp_, in the synthesized 60 ml suspension (V) can be calculated as follows (based on GR_SO4_ formula: Fe(II)_4_Fe(III)_2_(OH)_12_SO_4_•8H_2_O, with molar mass, M(GR_SO4_) = 780 g/mol:[6]

$\mathbf{m}{\mathbf{(}\mathbf{GR}_{\mathbf{SO4}}\mathbf{)}}_{\mathbf{60ml}}\mathbf{=}\left( \frac{\mathbf{[Fe(II)}_{\mathbf{s}}\boldsymbol{]\times}\mathbf{V}_{\mathbf{60ml}}}{\mathbf{4}} \right)\boldsymbol{\times}\mathbf{M(GR}_{\mathbf{GRSO4}}\mathbf{)=0.546 g}$ *Eqn. 4*

For each experiment we used 1 ml GR suspension, i.e., 9.1 mg of GR_SO4_, that contain 72.4 µmol Fe(II)_tot_, of which 46.7 µmol is Fe(II)_s_ and 25.7 µmol is Fe(II)_d_.

*Calculation of mass and volume of Fe^0^ core and FeS shell in S-nZVI:* We assume that: (i) S-nZVI is spherical, with an average radius of 50 nm and a FeS shell thickness of 5 nm [7]. Hence, the radius of the iron core (Fe^0^) is 45 nm. We can then calculate the relative volume % of the Fe^0^ core (%V_Fe0_) and the FeS shell (%V_FeS_) as follows:

$\frac{\mathbf{V}_{\mathbf{Fe0}}}{\mathbf{V}_{\mathbf{SnZVI}}}\mathbf{=}\frac{{\mathbf{(}\mathbf{r}_{\mathbf{Fe0}}\mathbf{)}}^{\mathbf{3}}}{{\mathbf{(}\mathbf{r}_{\mathbf{SnZVI}}\mathbf{)}}^{\mathbf{3}}}\mathbf{=0.73;}\boldsymbol{\%V}_{\mathbf{Fe0}}\mathbf{=0.73}\boldsymbol{\&}\boldsymbol{\%V}_{\mathbf{FeS}}\mathbf{=0.27}$ *Eqn. 5*

We used 2.6 mg of S-nZVI (m_tot-SnZVI_) for each experiment. Assuming the densities of Fe^0^ and FeS are as follows:

$$\delta_{Fe0}=7.87 g/{\mathrm{cm}^{3}}$$

$$\delta_{\mathrm{FeS}}=4.84 g/{\mathrm{cm}^{3}}$$

we can then calculate the average density of S-nZVI (δ_SnZVI_) and the total added S-nZVI volume (V_tot-SnZVI_) as follows:

$\boldsymbol{\delta}_{\mathbf{SnZVI}}\mathbf{=}\left( \boldsymbol{\%V}_{\mathbf{Fe0}}\boldsymbol{\cdot}\boldsymbol{\delta}_{\mathbf{Fe0}}\mathbf{+}\boldsymbol{\%V}_{\mathbf{FeS}}\boldsymbol{\cdot}\boldsymbol{\delta}_{\mathbf{FeS}} \right)\mathbf{=7.05}\mathbf{g}/{\mathbf{cm}^{\mathbf{3}}}$ *Eqn. 6*

$\mathbf{V}_{\mathbf{tot-SnZVI}}\mathbf{=}\frac{\mathbf{m}_{\mathbf{tot-SnZVI}}}{\boldsymbol{\delta}_{\mathbf{S-nZVI}}}\mathbf{=0.00037}\mathbf{cm}^{\mathbf{3}}$ *Eqn. 7*

Hence, the total added Fe^0^ volume, V_tot-Fe0_, the Fe^0^ mass, m_tot-Fe0_, and the Fe^0^ molar mass, n_tot-Fe0_, are:

$\mathbf{V}_{\mathbf{tot-Fe0}}\mathbf{=}\left( \boldsymbol{\%V}_{\mathbf{Fe0}}\boldsymbol{\cdot}\mathbf{V}_{\mathbf{tot-SnZVI}} \right)\mathbf{mm}^{\mathbf{3}}\mathbf{=2.7}\boldsymbol{\cdot10}^{\mathbf{-4}}\mathbf{cm}^{\mathbf{3}}$ *Eqn. 8*

$\mathbf{m}_{\mathbf{tot-Fe0}}\mathbf{=}\left( \mathbf{V}_{\mathbf{tot-Fe0}}\boldsymbol{\cdot}\boldsymbol{\delta}_{\mathbf{Fe0}} \right)\boldsymbol{= 2.12 \cdot}\mathbf{10}^{\mathbf{-3}}\mathbf{g=2.12 mg}$ *Eqn. 9*

$\mathbf{n}_{\mathbf{tot-Fe0}}\mathbf{=}\frac{\mathbf{m}_{\mathbf{tot-Fe0}}}{\mathbf{M(Fe)}}\boldsymbol{=3.80 \cdot}\mathbf{10}^{\mathbf{-5}}\boldsymbol{mol=38 \mu mol}$ *Eqn. 10*


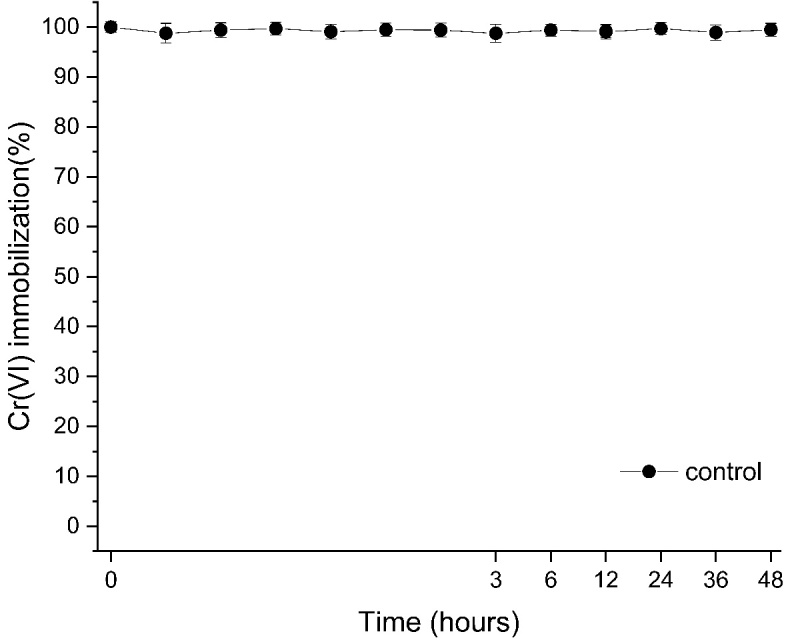


Figure S3: Control batch experiment with sand and Cr(VI) only (no added GR_SO4_ or S-nZVI) showed that Cr(VI) sorption to grain surfaces was negligible over 48 hours (note that x-axis is not linear).

# Text S3: NaNO_3_, Cr(VI) and total Cr measurements

The concentration of NaNO_3_ was determined spectrophotometrically at wavelengths of 240 nm. The concentration of Cr(VI) in collected effluent samples was determined by using two different spectrophotometric approaches: (i) 1,5-diphenylcarbazide colorimetric method (Environmental Protection Agency , EPA Method 7196A) and (ii) on-line measurements (at resolution < 1 s) using a UV-Vis flow through cell (2 mm pathlength, Hellma, Germany) connected with an UV-Vis spectrophotometer (STS-UV-L-50-400-SMA, 190-600 nm, Ocean Optics). The former approach is the most common method used for Cr(VI) determination but the latter one allows faster and inexpensive measurements.

1. For the colorimetric method, we used 0.5 mL of 1,5-diphenylcarbazide reagent (0.5 g 1,5-diphenylcarbazide in 100 mL acetone) and 25 ml of 0.01 M HCl solution to prepare the samples. Pink colour developed after approximately 15 min and absorbance was measured immediately at 540 nm using a 5 cm optical glass cell with a Perkin-Elmer Lambda 2S UV-Vis spectrophotometer (Waltham, MA, USA), calibrated using a four-point calibration curve.
2. Aqueous solutions of hexavalent chromium (without any complexing agents) absorb light in the ultraviolet and violet regions of the spectrum but wavelength-specific absorbance varies with solution pH and thus on the Cr(VI) speciation [8]. Initial calibrations showed that Cr(VI) concentration correlates linearly with absorbance at wavelengths of 274.3 and 371.3 nm at solution pH 7 and 9.5, and at wavelengths of 257 and 345 nm at pH 4.5 (Fig. S3a). Fig. S3b shows the calibration curves used for Cr(VI) aqueous solution (0.125-2 mmol/L) at pH 7. Our observations are in agreement with the study by Fournier-Salaün and Salaün (2007), where the authors reported that the absorption spectrum presents two maxima at 275 nm and at 371 nm in basic media and at 257 nm and at 349 nm in acid media. This on-line method allowed for collection of more accurate Cr(VI) measurements at a much higher temporal resolution compared to the method discussed above. Furthermore, it allowed us to monitor if any pH changes occur during the Cr(VI) injection.


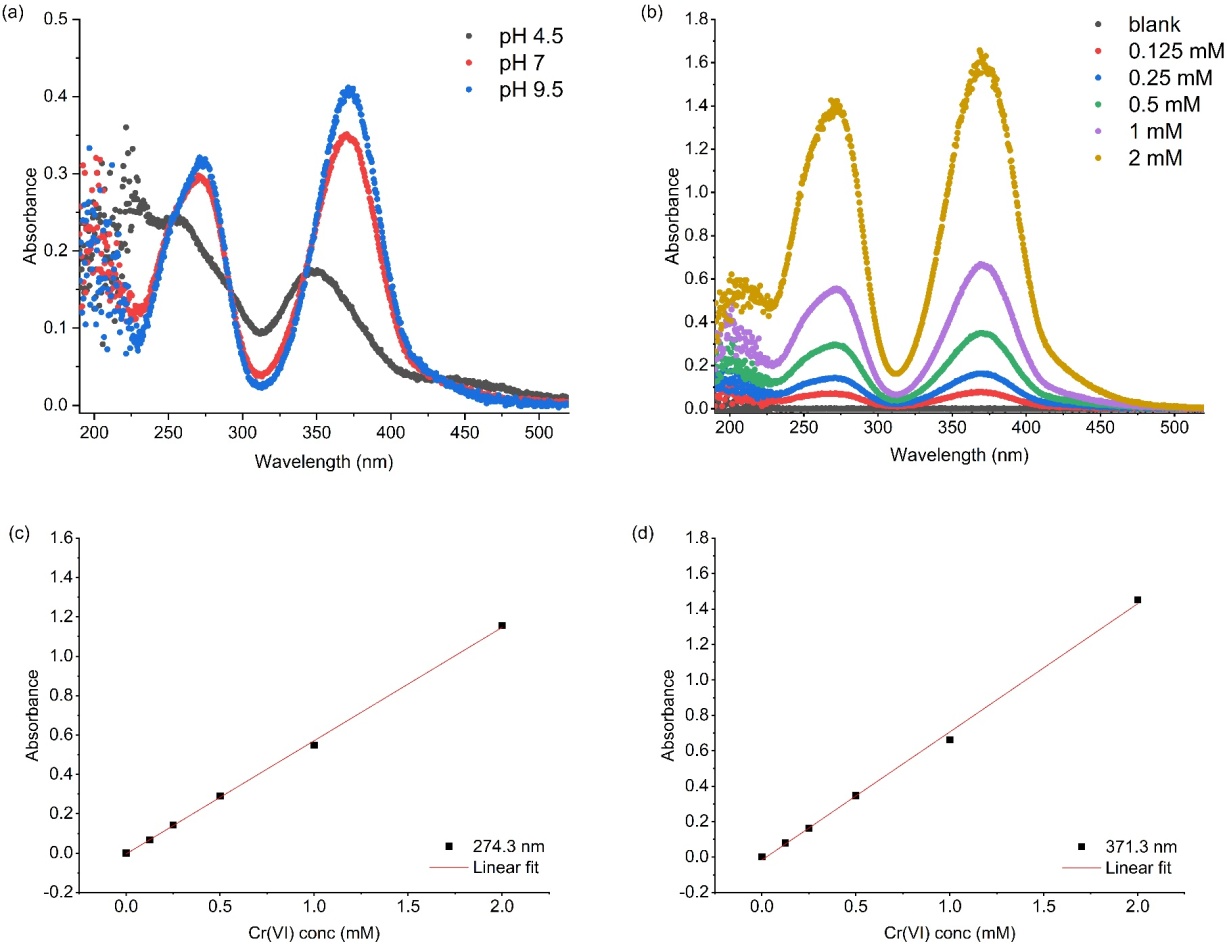


Figure S4: (a) Effect of Cr(VI) solution pH (4.5, 7.0 and 9.5) on UV-Vis absorption spectra; (b) UV-Vis spectra of Cr(VI) solutions of varying concentration (0.125 and 2 mM; at pH 7) used to make the calibration curves shown in (c) and (d) where adsorption readings were taken at 274.3 (R2 = 0.999) and 371.3 nm (R2 = 0.998), respectively.

**Additional details relevant to the results and discussion**


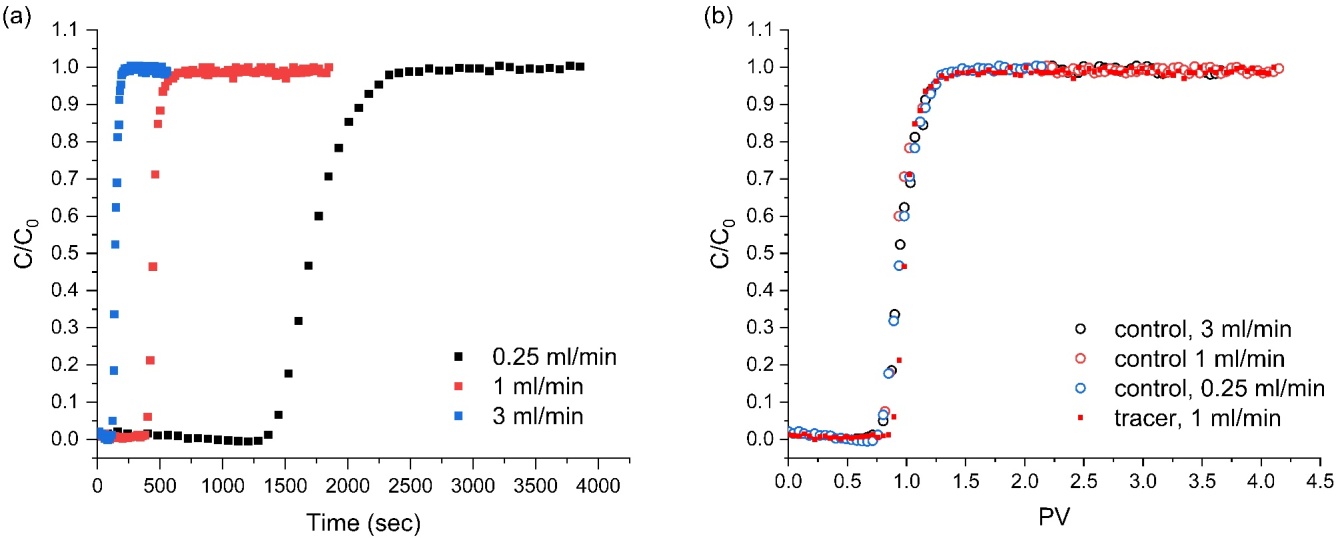


Figure S5: (a) BTCs (as a function of time) obtained by injecting 0.4 M NaNO3 at 0.25, 1 and 3 ml/min. (b) Comparison of BTCs (as a function of pore volume) obtained by injecting 0.5 mM Cr(VI) solution (control) at 0.25, 1 and 3 ml/min and 0.4 M NaNO3 solution (tracer) at 1 ml/min. All BTCs show no delay in breakthrough, i.e., are typical of non-reactive solutes.

*
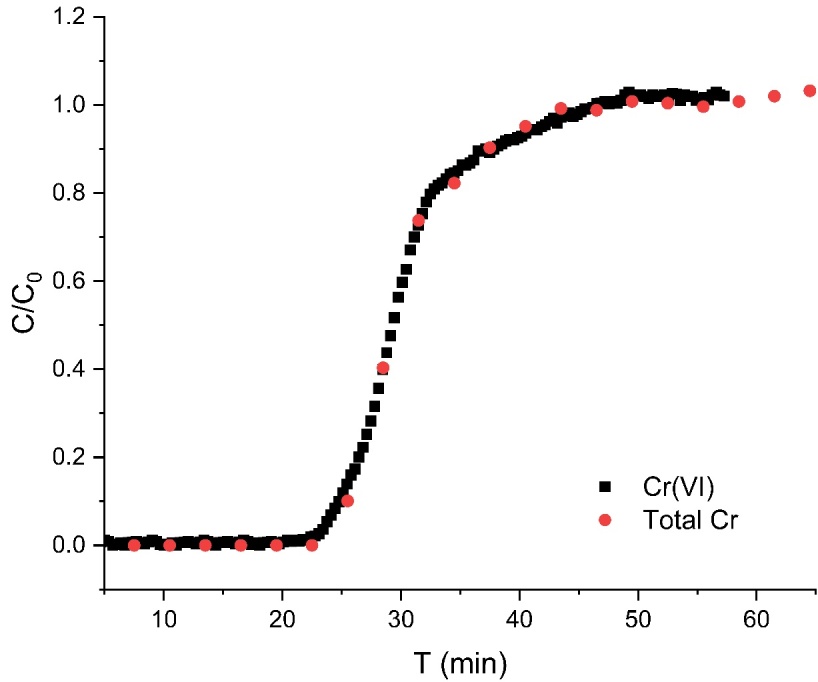
*

Figure S6: Comparison of BTCs obtained by measuring effluent Cr(VI) concentrations using the on-line UV-Vis set-up (black symbols) and by determining total Cr concentrations via ICP-OES in manually collected samples (red symbols). The test conditions were identical with [Cr(VI)0] = 0.5 mM, pH = 7 and flow rate = 1 ml/min. The fact that the two BTCs overlap demonstrates that Cr(VI) is the only Cr species detected in the effluent. Thus, any Cr(III) forming due to reduction is immobilised within the sand column.


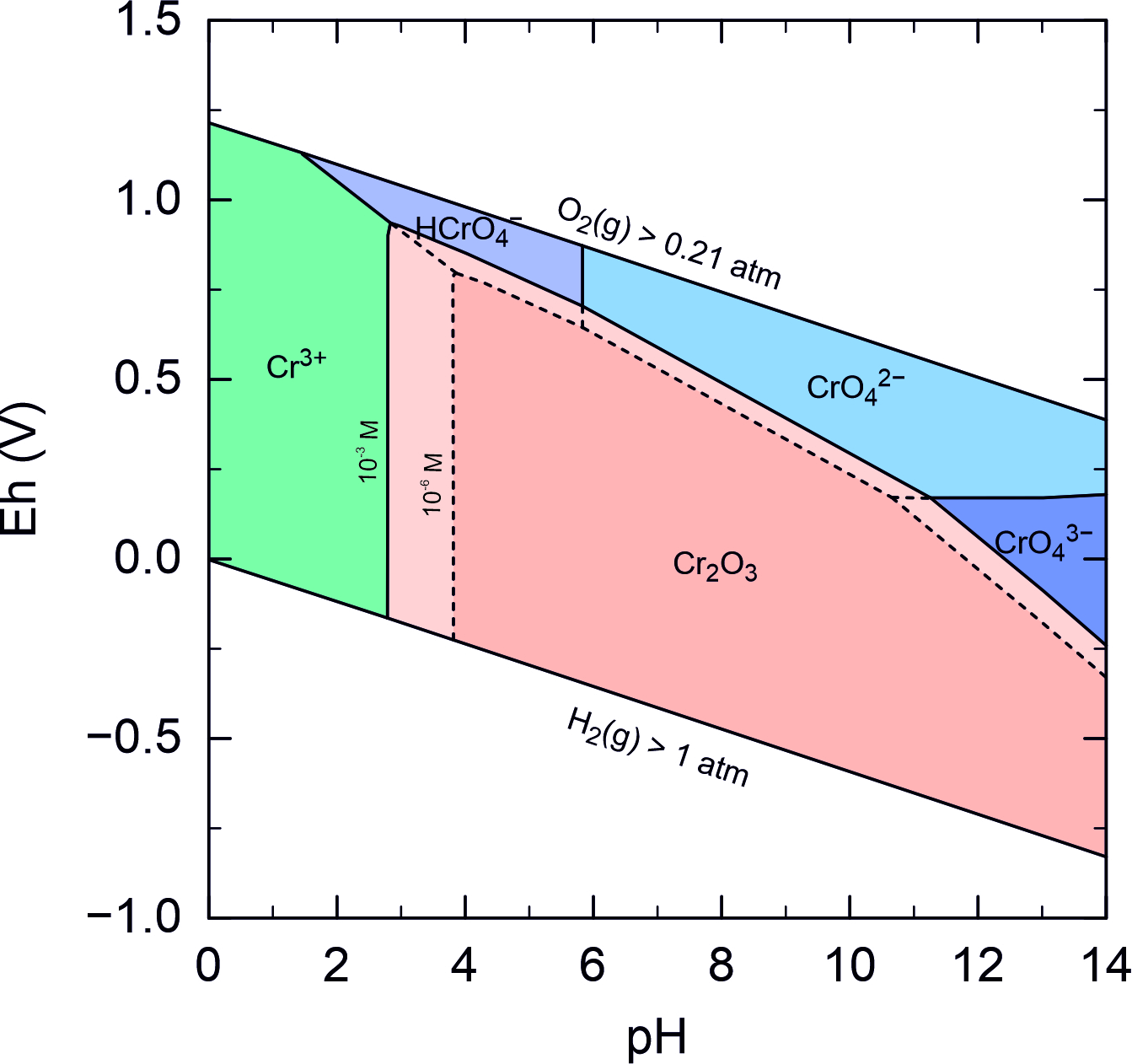


Figure S7 Eh-pH diagram for chromium based on experimental chromium concentration (10^-3^ M). Dashed line is based on lower concentrations (10^-6^ M). Calculations were made using PHREEQC (USGS).


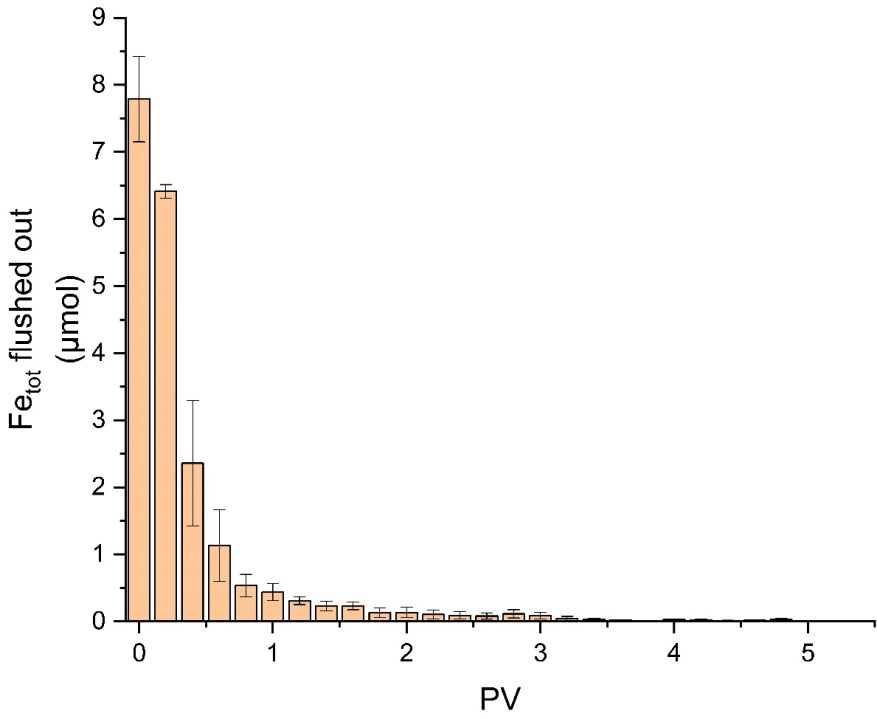


Figure S8: Prior to each Cr(VI) injection, columns were pre-flushed with 5 PVs of MilliQ water to remove aqueous Fe^2+^ present in the GR slurry that was mixed with the sand. Manually collected samples were analysed via ICP-OES to determine loss in total Fe as a function of flushed MilliQ pore volumes, which is depicted here. The plotted data show average values of 6 columns (3 columns run at 1 ml/min and 3 run at 3 ml/min).


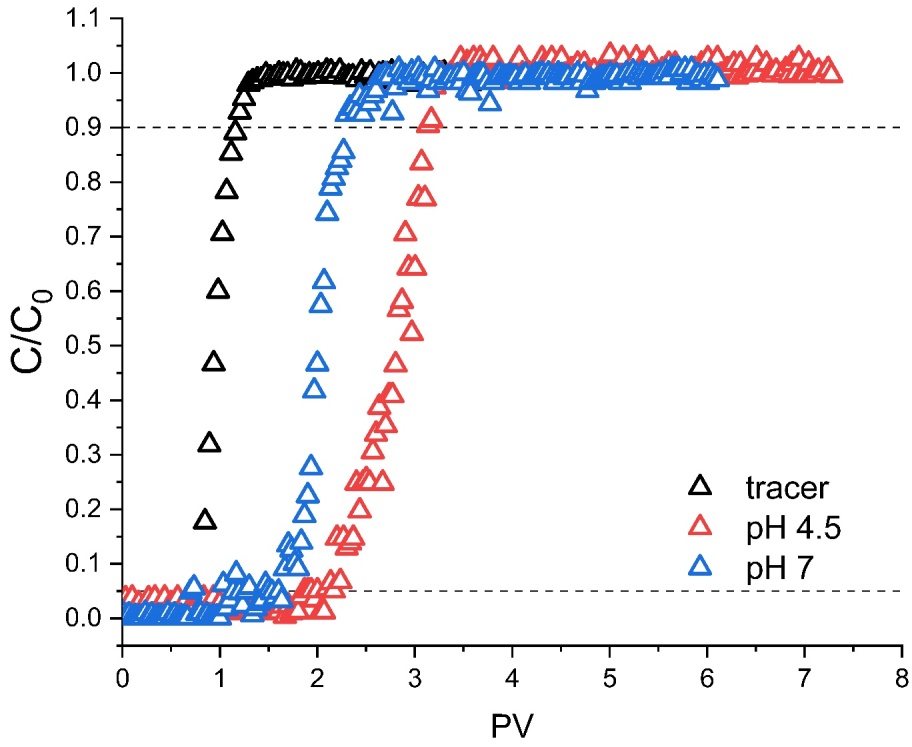


Figure S9 Cr(VI) breakthrough curves in S-nZVI amended packed sand columns as a function of inlet solution pH: 4.5 and 7.0 ([(Cr(VI)_0_]= 0.25 mM, flow rate= 0.25 ml/min). The dashed lines help to identify the breakthrough (C/C_0_=0.05) and exhaustion (C/C_0_=0.9) points on each curve.

Table S1 Breakthrough (C/C_0_= 0.05) and exhaustion (C/C_0_= 0.9) points expressed in terms of pore volumes (PV) for GRSO4 and S-nZVI sand column experiments performed at different [Cr(VI)_0_], flow rates and solution pHs (data taken from Fig. 4a, 5 and 6b).

| **Reactant type** | **Q (ml/min)** | **pH** | **[Cr(VI)_0_] (mM)** | **Breakthrough point (PV)** | **Exhaustion point (PV)** |
| --- | --- | --- | --- | --- | --- |
|  |  | 7.0 | 0.125 | 11 | 19 |
| GR_SO4_ | 0.25 | 7.0 | 0.25 | 5 | 8 |
|  |  | 7.0 | 0.5 | 4 | 5 |
|  |  | 7.0 | 1 | 1.5 | 2.5 |
|  |  | 7.0 | 0.125 | 11 | 19 |
|  |  | 7.0 | 0.25 | 5 | 8 |
| GR_SO4_ | 1 | 4.5 | 0.5 | 4.5 | 6 |
|  |  | 7.0 | 0.5 | 3 | 5 |
|  |  | 9.5 | 0.5 | 3 | 5 |
|  |  | 7.0 | 1 | 1.5 | 2.5 |
|  |  | 7.0 | 0.125 | 13 | 18 |
| GR_SO4_ | 3 | 7.0 | 0.25 | 5 | 8 |
|  |  | 7.0 | 0.5 | 3 | 5 |
|  |  | 7.0 | 1 | 1.5 | 2.5 |
|  | 0.25 | 4.5 | 0.25 | 2.2 | 3.5 |
|  | 0.25 | 7.0 | 0.25 | 1.5 | ~2 |
| S-nZVI | 1 | 7.0 | 0.25 | 1.5 | ~3 |
|  | 3 | 7.0 | 0.25 | 1.5 | ~3 |

# References

1. Thomas A, Eiche E, Göttlicher J, et al (2018) Products of Hexavalent Chromium Reduction by Green Rust Sodium Sulfate and Associated Reaction Mechanisms. Soil Syst 2:58

2. Williams AGB, Scherer MM (2001) Kinetics of Cr(VI) reduction by carbonate green rust. Environ Sci Technol 35:3488–3494

3. Bocher F, Géhin A, Ruby C, et al (2004) Coprecipitation of Fe(II-III) hydroxycarbonate green rust stabilised by phosphate adsorption. Solid State Sci 6:117–124

4. Guilbaud R, White ML, Poulton SW (2013) Surface charge and growth of sulphate and carbonate green rust in aqueous media. Geochim Cosmochim Acta 108:141–153

5. Eisele TC, Gabby KL (2014) Review of reductive leaching of iron by anaerobic bacteria. Miner Process Extr Metall Rev 35:75–105

6. Simon L, François M, Refait P, et al (2003) Structure of the Fe(II-III) layered double hydroxysulphate green rust two from Rietveld analysis. Solid State Sci 5:327–334

7. Mangayayam MC, Perez JPH, Dideriksen K, et al (2019) Structural transformation of sulfidized zerovalent iron and its impact on long-term reactivity. Environ Sci Nano 6:3422–3430

8. Fournier-Salaün MC, Salaün P (2007) Quantitative determination of hexavalent chromium in aqueous solutions by UV-Vis spectrophotometer. Cent Eur J Chem 5:1084–1093
